# Protective Factors against Morally Injurious Memories from the COVID-19 Pandemic on Nurses’ Occupational Wellbeing: A Cross-Sectional Experimental Study

**DOI:** 10.3390/ijerph191811817

**Published:** 2022-09-19

**Authors:** Mihaela Alexandra Gherman, Laura Arhiri, Andrei Corneliu Holman, Camelia Soponaru

**Affiliations:** Faculty of Psychology and Education Sciences, Alexandru Ioan Cuza University, Str. Toma Cozma 3, 700554 Iasi, Romania

**Keywords:** potentially morally injurious event (PMIE), turnover intentions, COVID-19 pandemic, basic psychological need satisfaction, nurses, burnout, episodic memories, self-determination theory, self-disclosure, perceived autonomy support

## Abstract

The COVID-19 pandemic was a fertile ground for nurses’ exposure to self- and other-Potentially Morally Injurious Events (PMIEs). Our study explored the effects of nurses’ memories of self- and other-PMIEs on occupational wellbeing and turnover intentions. Using an experimental design on a convenience sample of 634 Romanian nurses, we tested a conceptual model with PLS-SEM, finding adequate explanatory and predictive power. Memories of self- and other-PMIEs were uniquely associated with work engagement, burnout, and turnover intentions, compared to a control group. These relationships were mediated by the three basic psychological needs. Relatedness was more thwarted for memories of other-PMIEs, while competence and autonomy were more thwarted for memories of self-PMIEs. Perceived supervisor support weakened the indirect effect between type of PMIE and turnover intentions, through autonomy satisfaction, but not through burnout. Self-disclosure weakened the indirect effect between type of PMIE and turnover intentions, through autonomy satisfaction, and both burnout and work engagement. Our findings emphasize the need for different strategies in addressing the negative long-term effects of nurses’ exposure to self- and other-PMIEs, according to the basic psychological need satisfaction and type of wellbeing indicator.

## 1. Introduction

The fourth wave of the COVID-19 pandemic had a dramatic impact in Romania, with unprecedentedly high infection rates, registering the second highest per capita COVID-19 death rate in the world in October 2021 [1]. With one of the most underdeveloped healthcare systems in the European Union regarding infrastructure, sufficient staffing, and financing [2], the Romanian healthcare system quickly became overwhelmed [3,4]. Mirroring the global trend, nurses’ exposure to PMIEs spiked during this pandemic in Romania, with severe consequences on their wellbeing and psychological health, including—but not restricted to—moral injury [5,6,7,8,9].

Potentially Morally Injurious Events (PMIEs) are events which imply “perpetrating, failing to prevent, bearing witness to, or learning about acts that transgress deeply held moral beliefs and expectations” [10] (p. 697). Self-PMIEs are moral transgressions enacted under perceived external coercion, while other-PMIEs are moral transgressions to which the person assists without speaking/acting out, despite feeling as if they should. Exposure to PMIEs was associated with poorer COVID-19 psycho-social functional improvement over time in healthcare providers, especially for self-perpetrated PMIEs [11]. One potential explanation for this trend is the negative impact of the repeated recall of autobiographical episodic memories of these events. Thus, studies found that nurses’ episodic memories of PMIEs can have a unique negative association with their burnout, work motivation, work satisfaction, and adaptive performance several months after the event, mediated by autonomy thwarting [12]. Memories of self-PMIEs had stronger associations with burnout and turnover intentions compared to memories of other-PMIEs (i.e., enacted vs. witnessed PMIEs), mediated by the thwarting of all three basic psychological needs [13]. However, it remains unclear whether both memories of self-PMIEs and other-PMIEs would be associated with more burnout and turnover intentions when compared to a control group. This would be important to ascertain because it would contribute to our currently limited understanding of the distinctive harmful outcomes of exposure to self- and other-PMIEs [5,14,15,16]. Thus, our first aim was to experimentally investigate these differential associations with nurses’ occupational wellbeing and turnover intentions, mediated by the thwarting of all three basic psychological needs [17].

To date, protective factors against the negative effects of autobiographical episodic memories on psychological health and wellbeing have not been investigated, to the best of our knowledge. Previous findings suggest that nurses’ memories of PMIEs from the COVID-19 pandemic may not have yet been integrated in their autobiographical knowledge, an integration which might dramatically affect their work identities, behavior, and psychological health, with potential consequences on the healthcare organizational system [13]. Thus, departing from the mediating factors proposed, we set out to explore two potential moderators for the impact of memories of self- and other-PMIEs on burnout, turnover intentions, and work engagement. Perceived supervisor support and self-disclosure were assessed as moderators for autonomy and, respectively, relatedness thwarting.

### 1.1. Episodic Memories of PMIEs

Although correlated, self-PMIEs and other-PMIEs are distinct concepts, affecting psychological health differently [5,15,16]. However, this differential impact is still controversial. Thus, one study found that both were associated with increased depressive symptomatology in healthcare workers, but only self-PMIEs were associated with increased anxiety, PTSD, burnout, and disengagement [5]. Another study indicated that both types of PMIE could be associated with higher burnout, higher depressive symptoms, and worse quality of life in healthcare workers during the COVID-19 pandemic [14].

According to Self-Determination Theory (SDT), the three basic psychological needs are autonomy (i.e., the need to feel volitional and authentic in actions), competence (i.e., the need to feel effective and efficacious), and relatedness (i.e., the need to feel mutual connectedness and caring) [17]. In a previous study we conducted, our findings suggested that nurses’ work-related, autobiographical episodic memories of PMIEs during the COVID-19 pandemic had unique associations with increased burnout and, respectively, decreased work motivation, work satisfaction, and adaptive performance compared to their memories of severe moral transgressions (SMTs) [12]. In this study, we used SMTs as a control group, because they are similar to PMIEs in perceived moral severity, but different in terms of perceived external coercion. Thus, one of the defining characteristics of PMIEs is that they are moral transgressions perpetrated/witnessed against the person’s will (e.g., a nurse who prioritizes a younger patient over an older patient, based on directives according to which age is an indicator of odds of survival, and against their moral and professional ethical code, which would lead them to prioritize according to how critical the patient’s condition was) [5,6,7,8,9,10,11,12]. In contrast, SMTs are more similar to medical errors, in that the outcome of the transgression is very harmful (i.e., high in moral severity) [18,19,20], but the transgression is enacted in the absence of perceived external pressures (i.e., a nurse who chooses to come to work even if they are aware of being infected with the new coronavirus and spreads the disease to their patients) [20]. The differential associations of PMIEs and SMTs with the outcomes specified above were mediated by the extent to which nurses’ autonomy was thwarted in the two memories. Memories of PMIEs were associated with higher autonomy thwarting than memories of SMTs, which, in turn, was associated with more negative psychological health outcomes. However, we did not distinguish between self- and other-PMIEs in this study, treating PMIEs as a singular construct. We also did not explore the mediational role of the other two basic psychological needs (i.e., competence and autonomy) in the differences between PMIEs and SMTs in burnout. We address both these aspects in the current study.

In a different study, we compared nurses’ memories of self-PMIEs to their memories of other-PMIEs during the COVID-19 pandemic and found that the former had a stronger association with increased burnout and turnover intentions, mediated by all three basic psychological needs [13]. Thus, memories of self-PMIEs were more autonomy- and competence-thwarting than memories of other-PMIEs, a difference which we attributed to the omission bias [19]. When people enact a moral violation, they judge it as more harmful and blameworthy than when they allow it to happen without interfering. Hence, when forced to perpetrate an immoral act, it is likely that nurses perceived they had less autonomy than when forced to passively witness one, to justify their immoral behavior to themselves [20]. With moral values being central to their professional identities [21], their competence was also more threatened during self-PMIEs, which constituted a more direct threat to their identity compared to other-PMIEs [20,22]. In contrast, relatedness was more thwarted in memories of other-PMIEs compared to self-PMIEs, because they represent acts of organizational betrayal, put in motion by their peers or superiors [5,15,16]. Other-PMIEs were shown to be perceived as signs of disrespect towards nurses and exclusion from medical decision making [23,24,25], while self-PMIEs represent more distal acts of betrayal [15,16]. However, in this research, we did not use a control group in our design, nor did we explore differences in work engagement. Investigating work engagement alongside burnout is important because, although they do not form a single construct, work engagement contributes to our understanding of occupational wellbeing by adding the dimension of studying the characteristics of normal and satisfactory activity to the more pathology-oriented dimensions of burnout [26,27].

To our knowledge, so far, there are no studies which compare nurses’ memories of self-PMIEs and other-PMIEs from the COVID-19 pandemic to SMTs (i.e., a control group) in terms of associations with occupational wellbeing, comprising work engagement, and burnout, and, respectively, turnover intentions. Given the mixed findings on the effects of exposure to self- and other-PMIEs [5,13,14,15,16], it could be that the impact of memories of other-PMIEs is either not different or smaller than the impact of SMTs, since the latter can be need-thwarting as well, especially in terms of competence. On the other hand, this impact could be greater, because they could be more autonomy-thwarting than memories of SMTs [12,13], since they constitute passive moral transgressions perpetrated under environmental constraint [15,16]. These perceived environmental constraints represent morally laden limitations imposed on nurses by peers/supervisors/legislators during the pandemic, which could lead to more intense feelings of being disconnected from others and uncared for by them [15,16], which could, in turn, translate into more relatedness thwarting for memories of other-PMIEs compared to SMTs. The differential need thwarting of these types of events has direct implications for the types of interventions necessary for addressing the deficits in occupational wellbeing and turnover intentions associated with the two types of memories [10,16], as well as for prevention and reparatory efforts, which may focus on improving certain protective factors, such as perceived supervisor support and self-disclosure.

### 1.2. Perceived Supervisor Support

Perceived supervisor support is an organizational factor shown to influence nurses’ work satisfaction, job performance, and turnover intentions [28,29,30,31]. When nurses believe their supervisors value their contributions and care about their wellbeing [28], they experience more autonomy and job satisfaction, have lower turnover intentions, and provide better patient care [30,32]. Then, higher perceived supervisor support could decrease the impact of memory-related autonomy thwarting on wellbeing and turnover intentions. Knowing that their supervisor is fair and generally supportive of their autonomy could help them restructure their PMIE memories upon repeated recall as caused by exceptional circumstances uncharacteristic for their workplace [33].

### 1.3. Self-Disclosure

Self-disclosure is the process through which people allow themselves to be known by others [34], helping them cope with traumatic events [35,36], and operating as a protective factor against suicidal behavior [37]. By boosting social support and belongingness, it also protected war veterans against suicidal ideation after exposure to PMIE [38]. As such, nurses with higher levels of self-disclosure share their PMIE-related experiences, increasing their sense of belonging and social support, which could mitigate the negative influence of relatedness thwarting on their wellbeing and turnover intentions.

### 1.4. Occupational Wellbeing and Turnover Intentions during the COVID-19 Pandemic

Nurses’ wellbeing and turnover intentions were dramatically impacted by the COVID-19 pandemic, with consequences on patient care and their health [39,40]. Exposure to PMIEs and subsequent moral injury have been associated with decreased wellbeing and increased turnover intentions in healthcare providers and other populations, especially since the beginning of the COVID-19 pandemic [16,41,42,43]. With severe negative consequences at the individual and the organizational levels, more in-depth investigation in this area is necessary [39,41,44].

Two central work-related wellbeing indicators are work engagement and burnout [45]. Work engagement is an emotional and cognitive state manifested in vigor, dedication, and absorption [46]. While nurses’ pandemic-related stress and worries about their own health led to lower work engagement [47], concerns about the wellbeing of patients predicted higher work engagement [48,49]. Consequently, since being exposed to a PMIE leads to feelings of guilt and shame, adversely affecting the self-concept, we can expect that concerns about oneself are stronger than for SMTs, especially due to the high autonomy thwarting in memories of PMIEs [12]. Thus, after perpetrating an SMT, morally upward counterfactuals help restore the person’s morally good self-concept [12], guiding their future actions in that direction [22]. This emphasizes reparatory actions toward the harmed patients, which should lead to higher work engagement. In contrast, memories of PMIEs are not followed by morally upward counterfactuals to the same extent due to higher autonomy thwarting, which blocks mental simulations of alternative future courses of action [12,22]. As such, it is likely that work engagement is lower in this case, since the focus of the concerns would be the person rather than the patients.

Burnout is a syndrome characterized by the constant experiencing of work-related stress, expressed through exhaustion, cynicism, negative work attitudes, and low professional efficacy [50]. Having soared among nurses during this pandemic, it was predicted by nurses’ memories of self- and other-PMIEs, through basic psychological need thwarting [12,13,51], which alone can have a negative effect on burnout for up to two years after the event [52]. Turnover intentions were found to be the strongest predictor for turnover behavior, designating a conscious and deliberate willfulness to leave the workplace [53]. They represent “the last in a sequence of withdrawal cognitions, a set to which thinking of quitting and intent to search for alternative employment also belong” [54] (p. 262). Already higher in nursing [44], they spiked during the COVID-19 pandemic [55], being associated with memories of self-and other-PMIEs in this population [13].

Previous research suggested that burnout and work engagement may be antecedents of nurses’ turnover intentions [56,57]. Burnout mediated the relationship between perceived organizational justice and respect, work values, fairness, appropriate recognition and compensation, and, respectively, turnover intentions [58]. These workplace characteristics were previously linked to low relatedness satisfaction (i.e., low respect and fairness), low autonomy satisfaction (i.e., low perceived organizational justice), and low competence satisfaction (i.e., low recognition and compensation) [17]. As such, burnout might mediate the relationships between need satisfaction and turnover intentions in our model [58]. Low autonomy was associated with a decreased work engagement in nurses, as they found their jobs less meaningful and felt less responsible for their work, which was associated with higher turnover intentions [56]. Work engagement also mediated the relationship between ethical leadership and decision authority, associated with lower competence and relatedness satisfaction [58], and, respectively, turnover intentions [56]: the more nurses felt connected to and respected by their leaders, and the more their merits were acknowledged fairly, the lower their turnover intentions [57]. As such, work engagement might mediate the relationships between need satisfaction and turnover intentions in our model [54,55,56,57,58].

### 1.5. Present Study

**Figure 1:**
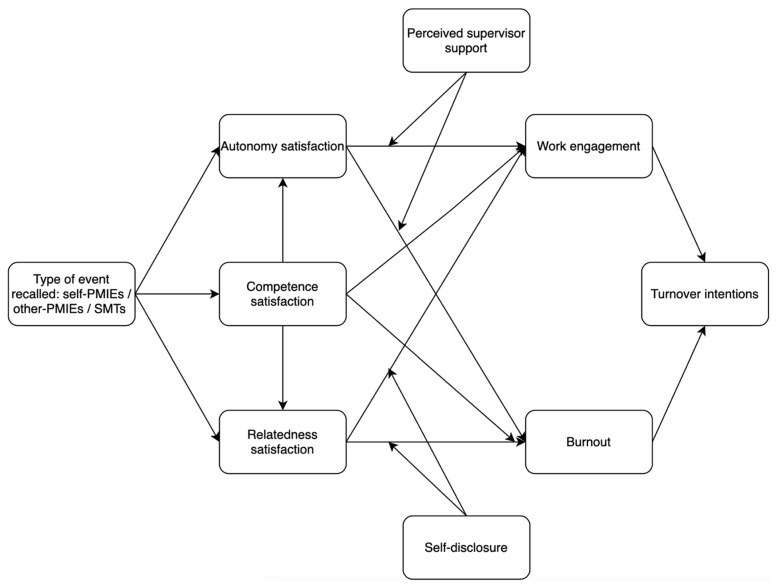
Conceptual model.

In our study, our first goal is to investigate whether other- and self-PMIEs may differently influence nurses’ occupational wellbeing and turnover intentions compared to SMTs, according to the thwarting of nurses’ basic psychological needs associated to each type of memory (Figure 1). As such, we hypothesized:

**H1:**  *Autonomy will be more thwarted in memories of self-PMIEs than in memories of other-PMIEs and in memories of SMTs*.

**H2:**  *Autonomy will be more thwarted in memories of other-PMIEs than in memories of SMTs*.

**H3:**  *Competence will be more thwarted in memories of self-PMIEs and, respectively, in memories of SMTs compared to memories of other-PMIEs*.

**H4:**  *Relatedness will be more thwarted in memories of other-PMIEs than in memories of self-PMIEs and in memories of SMTs*.

**H5:**  *Relatedness will be more thwarted in memories of self-PMIEs than in memories of SMTs*.

**H6:**  *Memories of self-PMIEs will be associated with lower work engagement, higher burnout, and more turnover intentions compared to memories of SMTs and other-PMIEs*.

**H7:**  *Memories of other-PMIEs will be associated with lower work engagement, higher burnout, and more turnover intentions compared to memories of SMTs*.

**H8:**  *Autonomy, competence, and relatedness thwarting will mediate the differences in burnout, turnover intentions, and work engagement between memories of self-PMIEs, other-PMIEs, and SMTs*.

Our second aim was to investigate whether self-disclosure and perceived autonomy support may operate as protective factors against the influence of basic psychological need thwarting on nurses’ occupational wellbeing and turnover intentions. Thus, we hypothesized:

**H9:**  *Nurses with higher levels of perceived autonomy support would experience lower burnout and turnover intentions, and, respectively, higher work engagement, when their memories are highly autonomy-thwarting, compared to nurses with lower levels of perceived autonomy support*.

**H10:**  *Nurses with higher levels of self-disclosure would experience lower burnout and turnover intentions, and, respectively, higher work engagement, when their memories are highly relatedness-thwarting, compared to nurses with lower levels of perceived autonomy support*.

**H11:**  *Burnout and work engagement will mediate the relationships between the types of events recalled, competence, relatedness, and autonomy satisfaction, and, respectively, turnover intentions*.

## 2. Materials and Methods

The manuscript complies with STROBE reporting guidelines for observational research [59].

### 2.1. Participants

A convenience sample of 634 Romanian nurses working in hospitals selected through snowballing techniques participated in our study, conducted in February 2022. We collected our data after the fourth wave of the COVID-19 pandemic hit Romania, with a devastating effect on the healthcare system. Infection rates soared to over 20,000 daily new cases, while mortality peaked at 500 deaths per day, in a country with 19 million inhabitants [60]. Given the unpreparedness of the medical system to handle this critical situation and relying on findings from previous waves in Romania [6], we anticipated that nurses in many specialties could have been exposed to PMIEs and attempted to include multiple specialties in our sample to boost representativeness.

First, we invited 608 nurses to participate to our research via e-mail and/or phone, and to extend the invitation to peers who met the inclusion criterion: having worked as a nurse in a hospital for more than six months during the COVID-19 pandemic. We obtained their contact information during previous data collection stages for other studies, when participants agreed to be contacted again for this purpose. A total of 524 of them agreed to participate, and another 241 nurses responded to the invitation extended by their peers, per our request. We randomized the 765 in the three experimental conditions and sent them links to Google Forms. Overall, 52 participants withdrew from the study, and 15 were eliminated because they failed the attention check. Another 64 participants from the self- and other-PMIE conditions were eliminated because their recalled events did not constitute PMIEs, according to their scores on the Moral Injury Events Scale. Hence, our final sample comprised 634 nurses, with 235 in the control group (37.06%), 214 in the other-PMIE condition (33.75%), and 185 in the self-PMIE condition (29.19%), from varied specialties (Table 1).

We proceeded in accordance with the ethical rules stipulated in the Declaration of Helsinki. We were granted approval by the ethics committee of our faculty. All participants were legally adults and consented to take part in our study. They were told that their participation was voluntary, and that dropout was possible at any stage. They were informed about anonymity and confidentiality of the information they provided. Due to the delicate nature of the information requested from them, we let them know that none of their data would be shared publicly or seen by any other third party, except for the first two authors. We decided to guarantee this aspect to our participants because they were reluctant to disclose the data needed for this study. In order to avoid social desirability in their responses, we offered them these guarantees. The information we gathered in this study remained securely stored for analyses only. Participants were rewarded for their willingness to take part in the study with five money prizes, each amounting to 100 RON, awarded to them based on a draw.

### 2.2. Procedure and Instruments

The study was self-paced. After reading and providing their informed consent, participants completed a series of socio-demographic items (i.e., age, sex, education, work experience, current specialty). Then, they filled in the measures of perceived supervisor support and self-disclosure.

*Perceived Supervisor Support* was measured with the six-item version of the Work Climate Questionnaire (WCQ) [61]. The final score (α = 0.933) was the sum of the individual scores, with answers provided on a seven-point Likert-type scale, from 1—“Strongly Disagree” to 7—“Strongly Agree” (e.g., “I feel that my manager provides me choices and options.”).

*Self-Disclosure* was measured with the 12-item Distress Disclosure Index (DDI; e.g., “When something unpleasant happens to me, I often look for someone to talk to.”) [62]. Answers were given on a five-point Likert-type scale, from 1—“Strongly disagree” to 5—“Strongly agree”. Alpha was 0.966.

Then, participants recalled episodic memories of self- and other-PMIEs and of SMTs, according to the experimental condition in which they had been randomly placed, after reading definitions and examples of the three constructs (see Appendix A for a detailed account of the experimental procedure). The nine-item Moral Injury Events Scale (MIES) modified to assess PMIEs among healthcare workers during the COVID-19 pandemic was filled in next [11]. (e.g., “I acted in a way that violated my own moral code or values in this instance.”). The scale was tested and used on Romanian healthcare staff [6]. Responses were provided from 1—“Strongly Agree” to 6—“Strongly Disagree”. To evaluate if memories constituted PMIEs, final scores were dichotomized, with answers of “Moderately Agree” to “Strongly Agree” on any of the nine items coded as exposure to a PMIE [11], eliminating participants not recounting PMIEs from the study.

Participants were also asked to morally judge the events recalled from 1—“Slightly Morally Wrong” to 7—“Very Morally Wrong” (“How morally wrong was your behavior in this instance?”) [22]. To check the manipulation of recalling SMTs and, respectively, PMIEs, we asked participants to rate the extent to which they perceived themselves as moral victims and transgressors in those circumstances in two items, with answers ranging from 1—“Not at All” to 7—“Very Much” [11]. To check the manipulation of recalling other-PMIEs and, respectively, self-PMIEs, we added an item to evaluate the degree to which participants perceived themselves as witnesses during the events on a scale ranging from 1—“Not at All” to 7—“Very Much”.

The level of need satisfaction experienced during their memories was self-rated, since previous research revealed a significant association between participants’ and independent judges’ ratings [63]. Each need was assessed through two items on a seven-point Likert scale, ranging from −3 (Strongly disagree) to +3 (Strongly agree), with 0—Do not agree nor disagree or not applicable: autonomy (e.g., “I felt free to do things and to think how I wanted”), competence (e.g., “I felt capable and skillful.”), and relatedness (e.g., “I felt connected to one or more people”) [63,64,65,66,67,68,69]. Individual scores for each need were averaged, with higher scores reflecting higher need satisfaction. Scores above zero indicated a need-satisfying memory, and scores under zero, a need-thwarting memory. The scale was used in previous research e.g., [12,13,52,63,64,65,66,67,68,69]. The reliability for the three scales was evaluated: Cronbach’s alpha for Autonomy = 0.922; Cronbach’s alpha for Competence = 0.862; and Cronbach’s alpha for Relatedness = 0.910.

Then, participants filled in the scales for burnout, turnover intentions, and work engagement. *Burnout* was measured with the Maslach Burnout Inventory, validated on the Romanian population [70]. The total score (α = 0.962) was the sum of the scores on the three dimensions evaluated: *emotional exhaustion* (EE; nine items; α = 0.985), *depersonalization* (DP; five items; α = 0.977), and *personal accomplishment* (PA; eight items; α = 0.986). Answers were given on a seven-point Likert-type scale, from 0—“Never” to 6—“Everyday”.

*Nurses’ turnover intentions* were evaluated with the three-item scale from the Michigan Organizational Assessment Questionnaire [71] (e.g., “I will probably look for a new job in the next year.”). Answers were given on a seven-point Likert-type scale, from 1—“Strongly disagree” to 7—“Strongly agree”. Alpha was 0.943.

*Work engagement* was measured with a shortened, nine-item version of the original Utrecht Work Engagement scale (UWES-9) [46]. The total score (α = 0.898) was the sum of the scores on the three dimensions evaluated: *vigor* (three items: e.g., “At work, I feel that I am bursting with energy.”; α = 0.954), *absorption* (three items: e.g., “I am immersed in my work.”; α = 0.959), and *dedication* (three items: e.g., “I am enthusiastic about my job.”; α = 0.957). Answers were given on a seven-point Likert-type scale, from 1—“Never” to 7—“Daily”.

We employed the attention check used by Stanley et al. [22]: “Do you feel that you paid attention, avoided distractions, and took the survey seriously?” Participants were informed that their answers would not influence their participation in the prize draw. Responses ranged from: 1—“No, I was distracted”; 2—“No, I had trouble paying attention”; 3—“No, I did not take this study seriously”; 4—“No, something else effected my participation negatively”; 5—“Yes”. We eliminated from our data analysis participants who responded 1, 2, 3, or 4.

## 3. Results

### 3.1. Data Analysis Strategy

First, we verified the validity of our experimental manipulations and participant randomization in the three experimental conditions with one-way and mixed repeated measures ANOVAs, and with chi-square tests of association. Second, socio-demographic differences in outcomes of interest were assessed with Welch’s independent sample t-tests and one-way ANOVAs, due to violations of the assumption of equal variances and unequal sample sizes (Table A2). For this purpose, we stratified “age” and “work experience”. Correlations between outcomes of interest were also computed. These data analyses were conducted in Jamovi 2 (The jamovi group, Sydney, Australia).

Second, we proceeded to assess our hypotheses, graphically depicted in the conceptual model presented in Figure 1. Since the type of event recalled was a categorical variable with three levels, we dummy-coded it and ran the analysis twice. According to our hypotheses, we first assessed differences between recalling other-PMIEs and recalling SMTs, and, respectively, between recalling self-PMIEs and recalling SMTs (i.e., SMTs were coded with 0, and self-PMIEs and other-PMIEs with 1). The second time we ran the model, we assessed differences between other-PMIEs and self-PMIEs and, respectively, SMTs and self-PMIEs (i.e., SMTs and other-PMIEs were coded with 1, while self-PMIEs with 0). Aside from these exogenous variables, our model included another two exogenous variables, respectively, the two moderators—self-disclosure and perceived supervisor support. Autonomy, competence, and relatedness satisfaction were included as mediators of the relationships between the type of event recalled and work engagement, turnover intentions, and burnout. In turn, the mediating role of work engagement and burnout on the relationship between need satisfaction and turnover intentions was also evaluated. Finally, our model tested whether self-disclosure moderated the effects of relatedness on work engagement, burnout, and turnover intentions, and, respectively, whether perceived supervisor support moderated the effects of autonomy on those three outcomes. We controlled for age and education level.

Work engagement and burnout are three-dimensional constructs. Treating multi-dimensional psychological constructs as reflective–reflective has been a contested practice, with newer perspectives suggesting that they should be considered reflective–formative second-order constructs [72]. Construct dimensions (i.e., first-order constructs) are considered reflective because their indicators (i.e., the items on the scales) can be removed or changed. By the same logic, if the second-order constructs were reflective–reflective, their dimensions could be removed or changed. However, this is not the case, because second-order constructs such as burnout and work engagement are formed by their dimensions; in line with previous research, we treated them as type II second-order reflective–formative constructs [73,74,75].

Given our reflective–formative constructs, average sample size, and non-normally distributed data for our endogenous constructs (work engagement: W = 0.99, *p* < 0.001; burnout: W = 0.98, *p* < 0.001; turnover intentions: W = 0.99, *p* < 0.001; autonomy: W = 0.97, *p* < 0.001; competence: W = 0.96, *p* < 0.001; relatedness: W = 0.98, *p* < 0.001), we assessed our model with partial least squares structural equation modelling (PLS-SEM), according to the recommendations of Becker et al. [76], Ringle et al. [77,78] and Hair et al. [79,80,81], and in line with previous research [73,74,75]. The minimum sample size needed for PLS-SEM does not depend on how complex the model is, but rather on the probability that the ratio of a path coefficient and its standard error is higher compared to the critical value of a test statistic for a given significance level [82]. Using the inverse square root method, and assuming a common power level of 80% and significance levels of 5%, the minimum sample size for our model would be 619, for minimum path coefficients of 0.05 to 0.1. The analyses were run in SmartPLS 4.0 [78]. Significance was assessed following a non-parametric bias-corrected and accelerated bootstrapping procedure with 10,000 subsamples [79,80,81].

First, we assessed our measurement model and examined factor loadings (>0.5), internal consistency (Cronbach’s Alpha > 0.7), composite reliability (rhoA and rhoC values > 0.7), convergent validity (AVE > 0.5), and discriminant validity (Fornell and Larcker criterion, HTMT, and cross-loadings) [79,80,81,83,84,85,86]. Our higher constructs were validated with the disjoint two-stage approach, an alternative to the repeated indicators approach, which was problematic for higher-order constructs [79,80,81,84,86]. First, we modelled our reflective lower-order constructs with their respective indicators, and all the relationships among them, except for moderations. Next, we modelled work engagement and burnout as formative constructs based on the latent scores obtained in the previous step for their respective dimensions, and we assessed multicollinearity with VIF values (<5) and outer weights and loadings based on significance (*p* < 0.001).

Second, we tested our hypotheses by evaluating our structural model with the PROCESS module in SmartPLS [87]. Similar to the PROCESS macro for SPSS, the PROCESS module in SmartPLS can be used for path analyses, as it estimates a set of equations with observed variables [88]. However, in SmartPLS, the indicators of a construct are automatically equally weighted [80]. We assessed direct, indirect, and moderating relationships, followed by conditional mediations, according to our hypotheses. Significance was assessed following a non-parametric bias-corrected and accelerated bootstrapping procedure with 10,000 subsamples [80].

The model’s predictive power was assessed with PLSpredict, a procedure used for out-of-sample prediction [89], which estimates the model on a training sample and assesses its predictive power on a holdout sample [90]. The root-mean-square error (RMSE) is usually employed to evaluate the degree of prediction error (i.e., differences between actual and predicted values). RMSE values are compared to naïve linear regression model benchmarks: if all PLS-SEM indicators have lower RMSE values compared to LM, then the model has high predictive power; if most PLS-SEM indicators have lower RMSE values compared to LM, then the model has medium predictive power; and if few PLS-SEM indicators have lower RMSE values compared to LM, then the model has low predictive power [90]. R^2^ and f^2^ coefficients (i.e., assessing the changes in R^2^ when any one predictor is excluded from the model) were examined to assess the explanatory power of our model, and Q^2^ was examined for predictive relevance.

### 3.2. Manipulation and Randomization Checks

To check our experimental manipulations, we ran a one-way and, respectively, a mixed repeated measures ANOVA to assess differences between the three experimental groups according to the perceived moral severity of the recalled PMIE, and, respectively, according to the perceived role in the event (i.e., witness, moral victim, moral perpetrator). Results showed no differences according to moral severity, as well as significant differences in terms of the three roles, supporting the validity of the experimental manipulation (Appendix B, Table A1).

To check participant randomization in the three experimental groups, we ran four one-way ANOVAs to assess differences in age, work experience, perceived supervisor support, and self-disclosure, and, respectively, two chi-square tests of association to assess differences in sex and in education levels. Results showed no significant differences for any of the participants’ characteristics (Appendix B, Table A1).

### 3.3. Participants’ Characteristics and Differences between Them according to Outcome Variables

Participants with less work experience (i.e., less than or equal to 10 years) had significantly lower *self-disclosure* than participants with more work experience (i.e., between 11 to 38 years). Similarly, younger participants (i.e., 21–30 years old) had lower self-disclosure compared to participants aged 41–57 years old (*t*(631) = −2.82, *p* = 0.014, *d* = −0.29) (Table A2).

Participants with less work experience (i.e., less than or equal to 10 years) experienced significantly less *work engagement* than participants with more work experience (i.e., between 11 to 36 years). Similarly, younger participants (i.e., 21–30 years old) experienced less work engagement compared to participants aged 31–40 years old (*t*(631) = −3.94, *p* < 0.001, *d* = −0.42) and compared to participants aged 41–57 years old (*t*(631) = −4.42, *p* < 0.001, *d* = −0.46). Participants with post-secondary studies experienced more work engagement than both participants with bachelor’s degrees (*t*(631) = 2.52, *p* = 0.032, *d* = 0.54), who experienced more work engagement than participants with master’s degrees (*t*(631) = 3, *p* = 0.008, *d* = 0.78) (Table A2).

Participants with less work experience reported more burnout than participants with more work experience. This trend was mirrored by the effects of participants’ age, with those aged 21 to 30 reporting more burnout than those aged 31 to 40 (*t*(631) = 2.57, *p* = 0.028, *d* = 0.27) and more than those aged 41 to 57 (*t*(631) = 4.17, *p* < 0.001, *d* = 0.43). We also looked at differences according to the three dimensions of burnout (EE, DP, PA) and found the same pattern of results for EE, with slight differences for PA and DP (Table A2).

*Turnover intentions* were stronger for the youngest participants (i.e., 21–30 years) than for their older counterparts (compared to 31–40 years: *t*(631) = 2.52, *p* = 0.032, *d* = 0.27; compared to 41–57 years: (*t*(631) = 2.39, *p* = 0.046, *d* = 0.25). Participants with bachelor’s studies had lower turnover intentions than participants with master’s degrees (*t*(631) = −2.47, *p* = 0.037, *d* = −0.64) (Table A2).

### 3.4. Correlational Analyses

Pearson’s correlations were computed to assess the associations between nurses’ wellbeing and turnover intentions, and, respectively, their self-disclosure, perceived supervisor support, and basic psychological need satisfaction in the recalled memories (Table A3). The higher the burnout, the higher the turnover intentions and, respectively, the lower their work engagement, perceived supervisor support, self-disclosure, and need satisfaction. The higher their work engagement, the lower their turnover intentions, and respectively, the higher their perceived supervisor support, autonomy, and relatedness satisfaction. The higher their turnover intentions, the lower their perceived supervisor support, self-disclosure, and need satisfaction. High perceived supervisor support was associated with high self-disclosure and competence satisfaction. Higher self-disclosure was associated with higher competence and autonomy satisfaction.

### 3.5. Measurement Model

In the first stage of the disjoint approach, we assessed the factor loadings, validity, and reliability of the model with lower-order constructs only. All factor loadings exceeded 0.5, so we kept all items (Table A4, Appendix D). Internal consistency reliability was satisfactory, with Cronbach’s alpha and rhoA values exceeding 0.7 (Table A4, Appendix D). Convergent validity was also satisfactory, with AVE values exceeding 0.50 (Table A4, Appendix D). Discriminant validity was established, according to the Fornell and Larcker [83] criterion (the square roots of AVE for all constructs were greater than their correlations with the other latent constructs—Table A5, Appendix D) and HTMT values (all values below 0.85—Table A5, Appendix D). Cross-loading analyses also showed that all indicators correlated more strongly with their own constructs compared to other constructs in the model.

In the second stage of the disjoint approach, we validated our two higher-order reflective formative constructs: burnout and work engagement. We checked the VIF values of the reflective–formative constructs, and they were below 5. Outer weights were significant, and outer loadings were above 0.5 (Table A6, Appendix D), according to the results of a 10,000-resample bootstrapping analysis.

### 3.6. Structural Model

To test our hypotheses, we ran two 10,000-sample bootstrapping analyses on unstandardized data, and we found that all R^2^ coefficients were larger than 0.10, which indicates that our proposed paths explained the variance of the endogenous constructs adequately [91], with contributions ranging from moderate to substantial [92] (Table A7, Appendix D). The f^2^ coefficients, indicative of effect sizes, ranged from high (0.438 for differences between the self-PMIE group and the control group in predicting autonomy) to negligeable (0.011 for differences between the self-PMIE group and the control group in predicting turnover intentions), in line with the complexity of the model [92] (Table A7, Appendix D). The Q^2^ values for our endogenous constructs were larger than 0, suggesting predictive relevance. The predictive power of the model was medium, with most PLS-SEM indicators having lower RMSE values compared to LM benchmarks, and with Q^2^ values for the indicators of our constructs above 0 (Table A8, Appendix D).

To test our first four hypotheses, we examined the paths from the type of event recalled to each of the three basic psychological needs (Table 2). Autonomy satisfaction was lower in memories of other-PMIEs (*M* = −0.75, *SD* = 1.09) compared to memories of SMTs (*M* = 0.24, *SD* = 1.16), and lower in memories of self-PMIEs (*M* = −1.59, *SD* = 1.08) than in memories of SMTs and other-PMIEs, confirming H1 and H2. Competence satisfaction was higher in memories of other-PMIEs (*M* = −0.75, *SD* = 1.06) compared to memories of SMTs (*M* = −1.5, *SD* = 1) and compared to memories of self-PMIEs (*M* = −1.49, *SD* = 0.9), confirming H3. Relatedness satisfaction was lower in memories of other-PMIEs (*M* = −1.35, *SD* = 1.16) compared to memories of SMTs (*M* = 0.19, *SD* = 1.19) and compared to memories of self-PMIEs (*M* = −0.67, *SD* = 1.1), but lower in memories of self-PMIEs than in memories of SMTs, confirming H4 and H5.

#### 3.6.1. Basic Psychological needs, Work Engagement, and Burnout Were Mediators between Type of Event Recalled and Turnover Intentions

We found significant indirect effects of type of event recalled on turnover intentions through the degree to which relatedness, autonomy, and competence were satisfied, work engagement, and burnout, confirming H8 and H11 (Table 3).

Thus, the differences in turnover intentions between memories of self-PMIEs (*M* = 14.9, *SD* = 3.19) and the control group (*M* = 12, *SD* = 3.32) could be explained by differences in autonomy and relatedness satisfaction, which were both associated with decreased work engagement for self-PMIEs (*M* = 23.4, *SD* = 6.7) compared to the control group (*M* = 36.4, *SD* = 7.87) and increased burnout for self-PMIEs (*M* = 88.1, *SD* = 22.6) compared to the control group (*M* = 55.3, *SD* = 17.6). The total effect of the differences in turnover intentions between memories of self-PMIEs and the control group was significant, but the direct effect was insignificant, suggesting full mediation.

The differences in turnover intentions between memories of other-PMIEs *M* = 13.2, *SD* = 3.49) and the control group (*M* = 12, *SD* = 3.32) could also be explained by differences in autonomy, relatedness, and competence satisfaction, which were all associated with decreased work engagement for other-PMIEs (*M* = 30.8, *SD* = 5.48) compared to the control group (*M* = 36.4, *SD* = 7.87), as well as increased burnout for other-PMIEs (*M* = 72.4, *SD* = 14.8) compared to the control group (*M* = 55.3, *SD* = 17.6). The total effect the differences in turnover intentions between memories of other-PMIEs and the control group was significant, but the direct effect was insignificant, suggesting full mediation.

The differences in turnover intentions between memories of other-PMIEs and self-PMIEs could be explained by differences in autonomy, which were associated with decreased work engagement and increased burnout for self-PMIEs compared to other-PMIEs. They were also explained by differences in competence, associated with increased burnout for self-PMIEs compared to other-PMIEs. However, differences in competence did not have a significant effect on work engagement which could explain the differences in turnover intentions, higher for self PMIEs than for SMTs. Concerning differences in relatedness between self- and other-PMIEs, they accounted for differences in work engagement (higher for other-PMIEs), which explained differences in turnover intentions (higher for self-PMIEs). However, differences in relatedness did not account for differences in burnout, explaining differences in turnover intentions for these two types of events recalled. As such, the mediation was partial. For the other mediations in the model, see Table A9 (Appendix D).

#### 3.6.2. Moderation Analyses

To test H9, we assessed the moderating role of perceived supervisor support on the relationships between autonomy satisfaction and burnout, autonomy satisfaction and work engagement, and, respectively, autonomy satisfaction and turnover intentions in our model. To test H10, we assessed the moderating role of self-disclosure on the relationships between relatedness satisfaction and burnout, relatedness satisfaction and work engagement, and, respectively, autonomy satisfaction and turnover intentions in our model. We were interested in assessing the rates of change in the indirect effects between the types of events recalled and turnover intentions at lower and higher levels of the two moderators (Table 4; for direct and interaction effects, see Table A10, Appendix D; for conditional direct effects and other conditional indirect effects in our model, please see Table A11 and, respectively, Table A12 in Appendix D).

To do this, we computed indices of moderated mediation [93].

**Figure 2:**
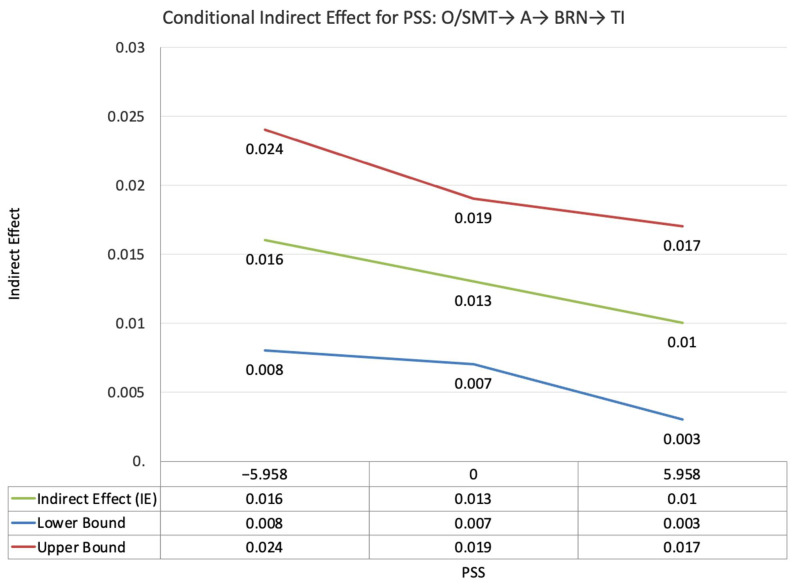
Conditional indirect effects of the difference between other-PMIEs compared to the control group on turnover intentions at low, average and high levels of perceived supervisor support, through autonomy satisfaction and burnout.

The index value of perceived supervisor support for the moderated mediation effect between other-PMIEs compared to the control group and turnover intentions, through autonomy satisfaction and burnout, was not statistically significant (ω = −0.003, *SE* = 0.004, T = −0.68, 95% CI = [−0.011, 0.003]). This suggests that, at higher levels of perceived supervisor support, the indirect effect of self-PMIEs compared to the control group was not statistically significantly lower compared to the indirect effect at low perceived supervisor support (Figure 2).

**Figure 3:**
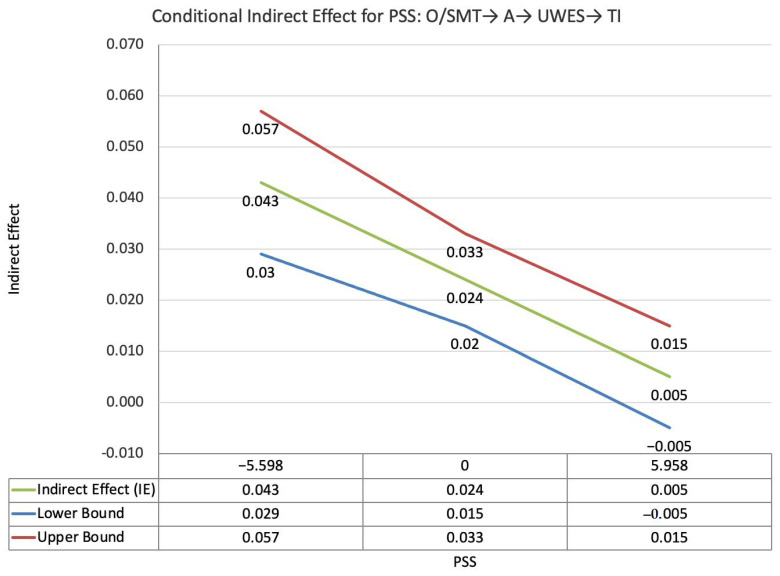
Conditional indirect effects of the difference between other-PMIEs compared to the control group on turnover intentions at low, average and high levels of perceived supervisor support, through autonomy satisfaction and work engagement.

The index value of perceived supervisor support for the moderated mediation effect between other-PMIEs compared to the control group and turnover intentions, through autonomy satisfaction and work engagement, was significant (ω = −0.019, *SE* = 0.008, T = −2.29, 95% CI = [−0.033, −0.007]). This suggests that, at higher levels of perceived supervisor support, the indirect effect of self-PMIEs compared to the control group was lower compared to the indirect effect at low perceived supervisor support. With an increase in perceived supervisor support, differences between other-PMIEs and the control group in turnover intentions were reduced (Figure 3).

**Figure 4:**
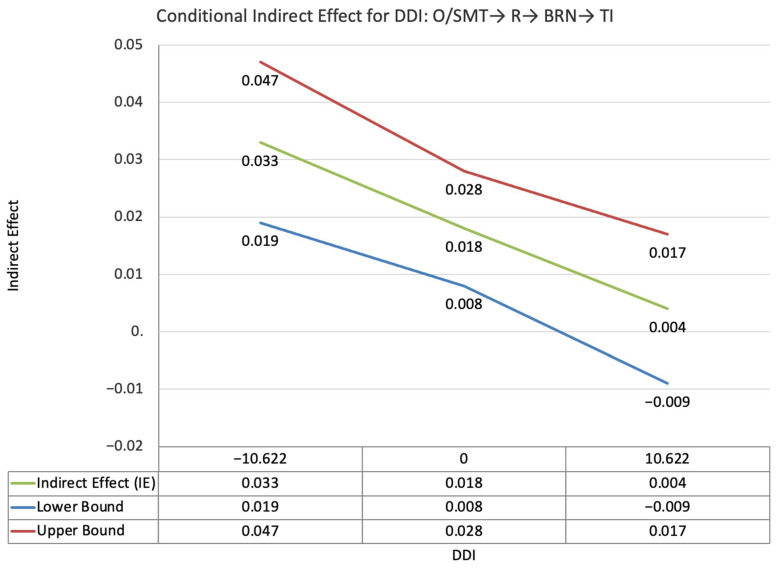
Conditional indirect effects of the difference between other-PMIEs compared to the control group on turnover intentions at low, average and high levels of self-disclosure, through relatedness satisfaction and burnout.

The index value of self-disclosure for the moderated mediation effect between other-PMIEs compared to the control group and turnover intentions, through relatedness satisfaction and burnout, was significant (ω = 0.028, *SE* = 0.02, T = 1.87, 95% CI = [0.04, 0.05]). This suggests that, at higher levels of self-disclosure, the indirect effect of self-PMIEs compared to the control group was lower compared to the indirect effect at low self-disclosure. With an increase in self-disclosure, differences between other-PMIEs and the control group in turnover intentions were reduced (Figure 4).

**Figure 5:**
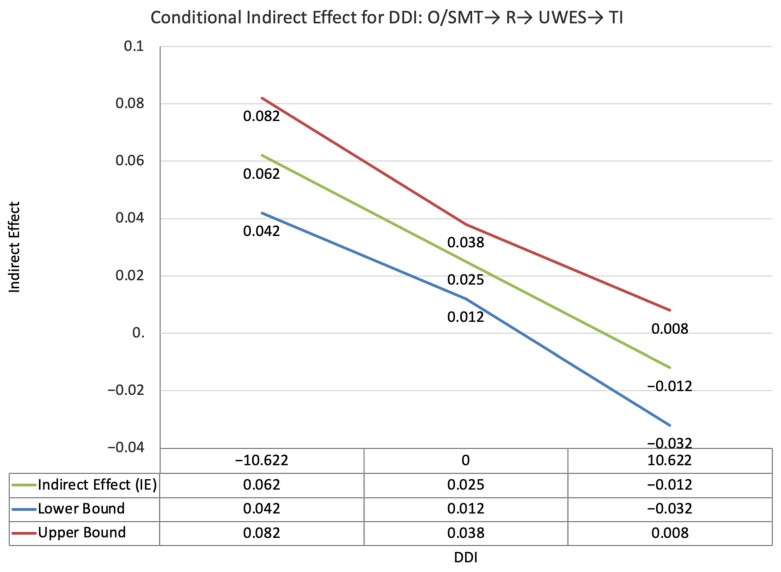
Conditional indirect effects of the difference between other-PMIEs compared to the control group on turnover intentions at low, average and high levels of self-disclosure, through relatedness satisfaction and work engagement.

The index value of self-disclosure for the moderated mediation effect between other-PMIEs compared to the control group and turnover intentions, through relatedness satisfaction and work engagement, was significant (ω = −0.018, *SE* = 0.007, T = −2.43, 95% CI = [−0.03, −0.007]). This suggests that, at higher levels of self-disclosure, the indirect effect of self-PMIEs compared to the control group was lower compared to the indirect effect at low self-disclosure. With an increase in self-disclosure, differences between other-PMIEs and the control group in turnover intentions were reduced (Figure 5).

**Figure 6:**
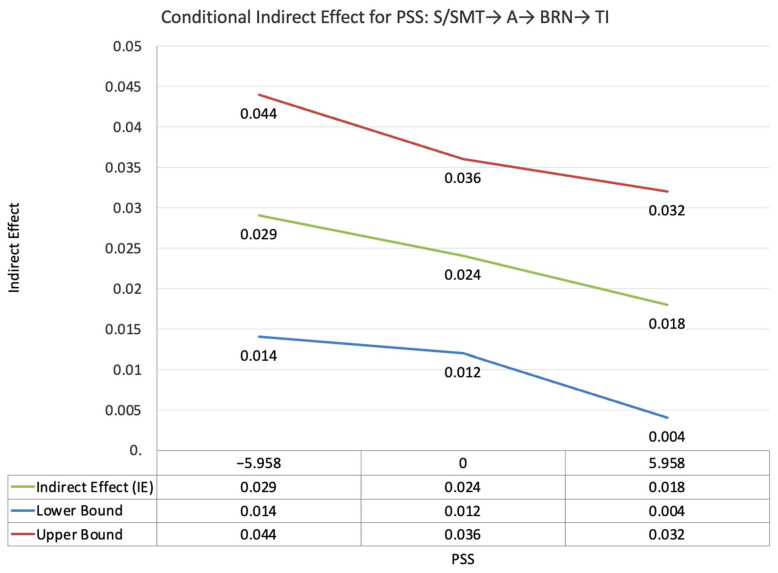
Conditional indirect effects of the difference between self-PMIEs compared to the control group on turnover intentions at low, average and high levels of perceived supervisor support, through autonomy satisfaction and burnout.

The index value of perceived supervisor support for the moderated mediation effect between self-PMIEs compared to the control group and turnover intentions, through autonomy satisfaction and burnout, was not statistically significant (ω = 0.006, *SE* = 0.008, T = 0.69, 95% CI = [−0.006, 0.02]). This suggests that, although at higher levels of perceived supervisor support, the indirect effect of self-PMIEs compared to the control group is slightly lower compared to the indirect effect at low perceived supervisor support, this difference does not reach statistical significance (Figure 6).

**Figure 7:**
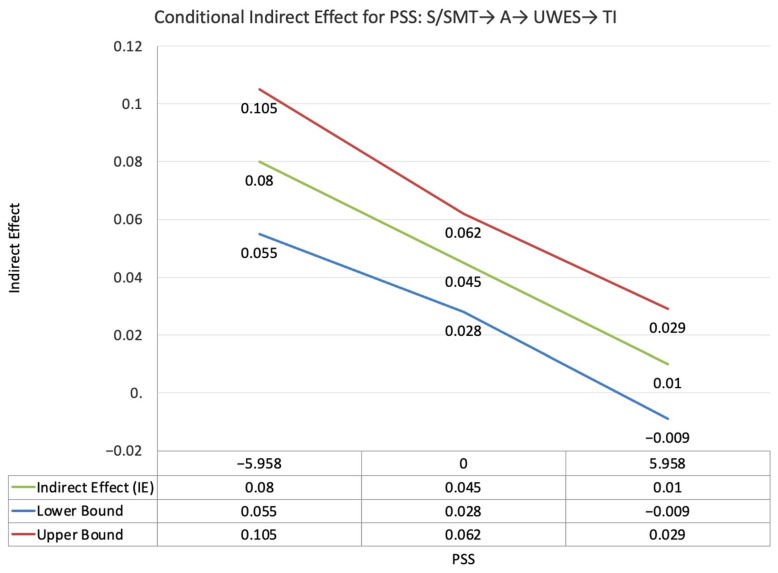
Conditional indirect effects of the difference between self-PMIEs compared to the control group on turnover intentions at low, average and high levels of perceived supervisor support, through autonomy satisfaction and work engagement.

The index value of perceived supervisor support for the moderated mediation effect between self-PMIEs compared to the control group and turnover intentions, through autonomy satisfaction and work engagement, was significant (ω = 0.035, *SE* = 0.015, T = 2.38, 95% CI = [0.013, 0.06]). This suggests that, at higher levels of perceived supervisor support, the indirect effect of self-PMIEs compared to the control group was lower compared to the indirect effect at low perceived supervisor support. With an increase in perceived supervisor support, differences between self-PMIEs and the control group in turnover intentions were reduced (Figure 7).

**Figure 8:**
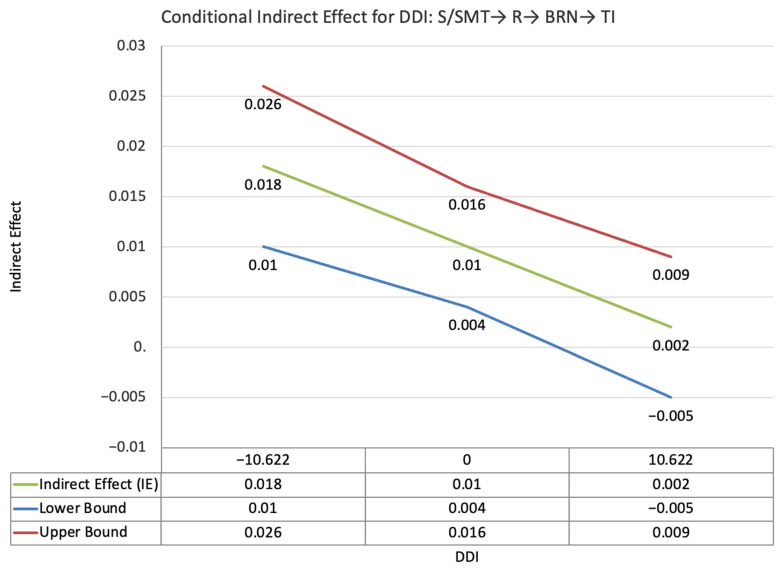
Conditional indirect effects of the difference between self-PMIEs compared to the control group on turnover intentions at low, average and high levels of self-disclosure, through relatedness satisfaction and burnout.

The index value of self-disclosure for the moderated mediation effect between self-PMIEs compared to the control group and turnover intentions, through relatedness satisfaction and burnout, was significant (ω = 0.009, *SE* = 0.006, T = 1.58, 95% CI = [0.001, 0.019]). This suggests that, at higher levels of self-disclosure, the indirect effect of self-PMIEs compared to the control group was lower compared to the indirect effect at low self-disclosure. With an increase in self-disclosure, differences between self-PMIEs and the control group in turnover intentions were reduced (Figure 8).

**Figure 9:**
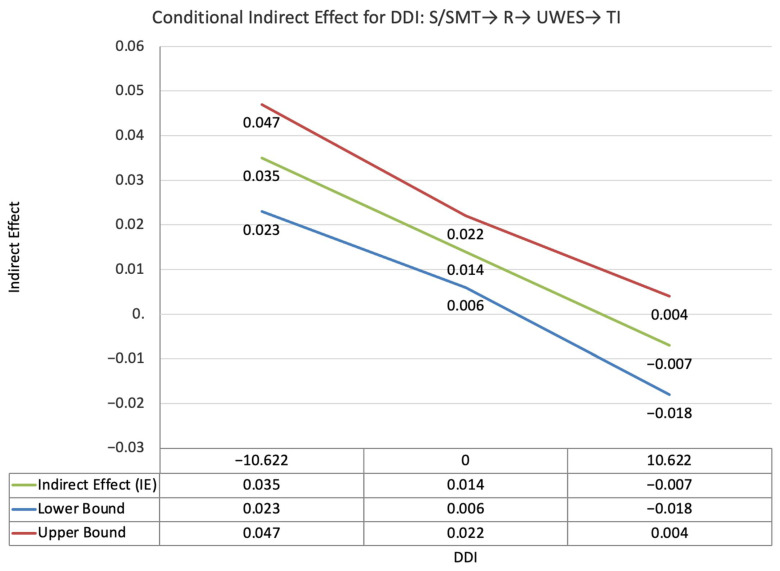
Conditional indirect effects of the difference between self-PMIEs compared to the control group on turnover intentions at low, average and high levels of self-disclosure, through relatedness satisfaction and work engagement.

The index value of self-disclosure for the moderated mediation effect between self-PMIEs compared to the control group and turnover intentions, through relatedness satisfaction and work engagement, was significant (ω = 0.023, *SE* = 0.01, T = 2.39, 95% CI = [0.009, 0.004]). This suggests that, at higher levels of self-disclosure, the indirect effect of self-PMIEs compared to the control group was lower compared to the indirect effect at low self-disclosure. With an increase in self-disclosure, differences between self-PMIEs and the control group in turnover intentions were reduced (Figure 9).

**Figure 10:**
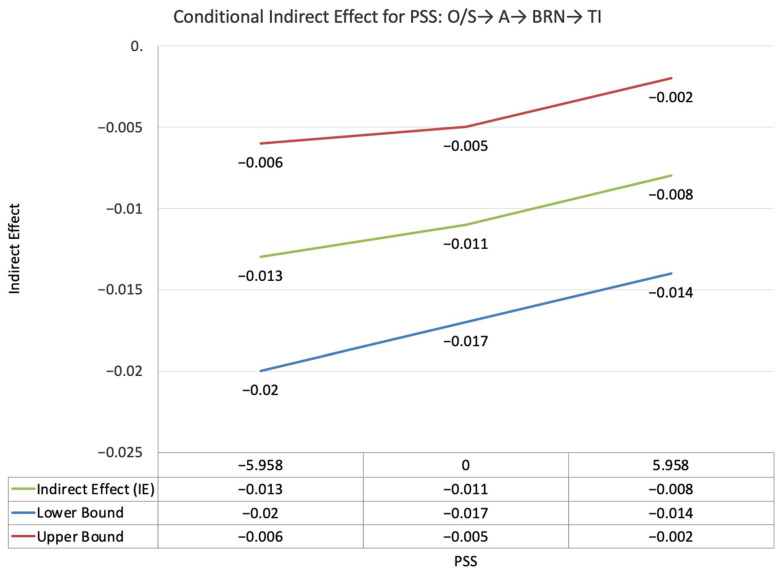
Conditional indirect effects of the difference between self-PMIEs compared to other-PMIEs on turnover intentions at low, average and high levels of perceived supervisor support, through autonomy satisfaction and burnout.

The index value of perceived supervisor support for the moderated mediation effect between self-PMIEs compared to other-PMIEs and turnover intentions, through autonomy satisfaction and burnout, was not statistically significant (ω = 0.003, *SE* = 0.004, T = 0.686, 95% CI = [−0.003, 0.009]). This suggests that, at higher levels of perceived supervisor support, the indirect effect of self-PMIEs compared other-PMIEs was not significantly lower compared to the indirect effect at low perceived supervisor support (Figure 10).

**Figure 11:**
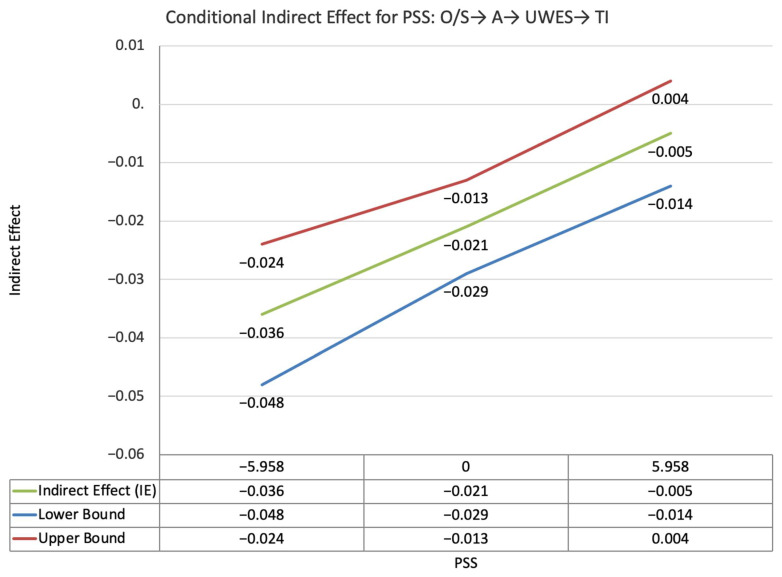
Conditional indirect effects of the difference between self-PMIEs compared to other-PMIEs on turnover intentions at low, average and high levels of perceived supervisor support, through autonomy satisfaction and work engagement.

The index value of perceived supervisor support for the moderated mediation effect between self-PMIEs compared to other-PMIEs and turnover intentions, through autonomy satisfaction and work engagement, was significant (ω = 0.016, *SE* = 0.007, T = 2.31, 95% CI = [0.006, 0.028]). This suggests that, at higher levels of perceived supervisor support, the indirect effect of self-PMIEs compared to other-PMIEs was lower compared to the indirect effect at low perceived supervisor support. With an increase in perceived supervisor support, differences between self-PMIEs and other-PMIEs in turnover intentions were reduced (Figure 11).

**Figure 12:**
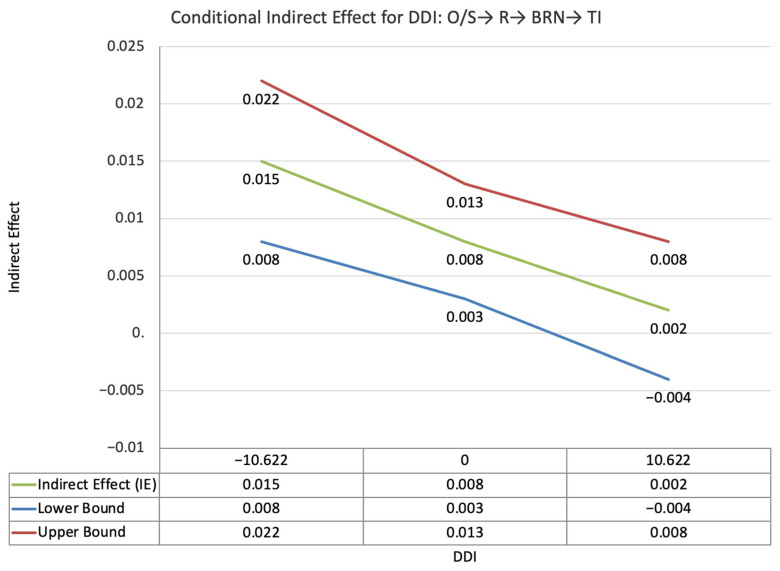
Conditional indirect effects of the difference between self-PMIEs compared to other-PMIEs on turnover intentions at low, average and high levels of self-disclosure, through relatedness satisfaction and burnout.

The index value of self-disclosure for the moderated mediation effect between self-PMIEs compared to other-PMIEs and turnover intentions, through relatedness satisfaction and burnout, was significant (ω = −0.024, *SE* = 0.013, T = −1.83, 95% CI = [−0.47, −0.004]). This suggests that, at higher levels of self-disclosure, the indirect effect of self-PMIEs compared to other-PMIEs was lower compared to the indirect effect at low self-disclosure. With an increase in self-disclosure, differences between self-PMIEs and other-PMIEs in turnover intentions were reduced (Figure 12).

**Figure 13:**
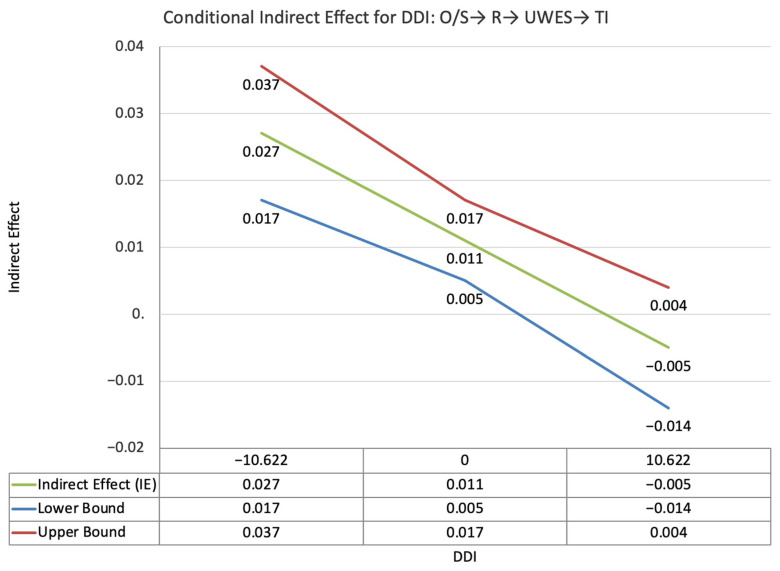
Conditional indirect effects of the difference between self-PMIEs compared to other-PMIEs on at low, average and high levels of self-disclosure, through relatedness satisfaction and work engagement.

The index value of self-disclosure for the moderated mediation effect between self-PMIEs compared to other-PMIEs and turnover intentions, through relatedness satisfaction and work engagement, was significant (ω = −0.018, *SE* = 0.007, T = −2.43, 95% CI = [−0.03, −0.007]). This suggests that, at higher levels of self-disclosure, the indirect effect of self-PMIEs compared to other-PMIEs was lower compared to the indirect effect at low self-disclosure. With an increase in self-disclosure, differences between self-PMIEs and other-PMIEs in turnover intentions were reduced (Figure 13).

## 4. Discussion

The Romanian healthcare system was the most affected one in Europe during the fourth wave of the COVID-19 pandemic, in terms of the disproportion between resources of all types (e.g., medical supplies, understaffing, insufficient time for patient care) and number of patients requiring medical attention [1,2,3,4]. Previous research showed that this constituted a fertile ground for PMIEs [5,6,7,8,9,10,11,12,13,14,15,16]. This study aimed to investigate the differential effects of Romanian nurses’ episodic memories of self- and other-PMIEs during the fourth wave of the COVID-19 pandemic compared to a control group on their occupational wellbeing and turnover intentions, according to basic psychological need thwarting, as well as two potential protective factors for these relationships: perceived supervisor support and self-disclosure. Building on previous studies comparing memories of self-PMIEs to other-PMIEs [13] and memories of undifferentiated PMIEs to memories of SMTs [12], we designed an experiment to better isolate the potential outcomes of nurses’ exposure to self-PMIEs from exposure to other-PMIEs, in line with past recommendations [5,13,14,15,16]. Our results partially supported our initial hypotheses. The differences in turnover intentions between nurses who recalled memories of self- and other-PMIEs, compared to each other and to the control group, were associated with significant differences in autonomy, competence, and relatedness, which, in turn, were associated with differences in burnout and work engagement. Higher levels of self-disclosure operated as a protective factor for burnout and work engagement, by weakening the strength of their association with relatedness satisfaction and with turnover intentions. Higher perceived supervisor support also helped weaken the association between autonomy satisfaction and work engagement, but it did not have the same effect on burnout in our sample.

The COVID-19 pandemic constituted an unprecedented crisis for healthcare systems all around the world, dramatically impacting patientcare and the psycho-social health and functioning of healthcare professionals. As frontline workers, nurses were among the most affected social categories, especially in terms of exposure to PMIEs [5,6,7,8,9,10,11]. This occurred because of the sudden ethical shift brought about by the pandemic: from nursing ethics, which include values from the patient-centered model of care, rooted in deontological ethics, to the public-health-centered approach, rooted in consequentialist ethics, adopted out of necessity during the pandemic [94,95,96]. As such, the transition from morally valuing the life of each patient to maximizing the number of lives saved led to ethical conflicts, amounting to PMIEs in many instances [5,6,7,8,9,10,11]. Aside from moral injury, exposure to PMIEs may have long-term consequences on a multitude of (occupational) health indicators, many of which remain uninvestigated and unaddressed by specific interventions [11,15,16]. Of these, the impact of the autobiographical episodic memories of self- and other-PMIEs should be of immediate concern, given that research suggests they might not have been integrated in autobiographical knowledge yet [13].

Autobiographical knowledge refers to semantic information about the self-concept, informing us about who we are and how we should act. Episodic memories of events which are in stark contrast with what we already know about ourselves are difficult to integrate into autobiographical knowledge, but they can guide behavior and shape attitudes prior to integration as well [64]. For nurses, integrating memories of PMIEs would change their self-concept and work identities, expanding the moral boundaries encompassing their schemas about patientcare and their roles in it [13]. If they internalize such “lessons” from the pandemic, according to which they and their peers are capable of, for instance, prioritizing resources according to arbitrary (and sometimes, discriminatory) criteria e.g., age; [95,96], acts that were once inconceivable outside of crisis situations become possible in more ordinary times. This could result in a catastrophic setback from the model of patient-centered care in nursing.

Aside from organizational and systemic consequences, nurses integrating such morally dissonant identity elements would lead to dehumanization, dissociation mechanisms, and a wide array of pathological outcomes [15,16], which could also contribute to decreasing the quality of patientcare. Thus, as our findings show, both memories of self- and other-PMIEs were associated with significant decreases in work engagement and, respectively, increases in burnout and turnover intentions, compared to a control group, outcomes which contribute to poorer job performance [97,98]. To prevent and treat these consequences of nurses’ exposure to self- and other-PMIEs, we first have to better distinguish the unique effects of each on occupational wellbeing and turnover intentions, and to identify the mechanisms through which the memories of these events negatively affect these outcomes. This would expand our search for potential paths of intervention, from reconstructing memories to addressing the basic psychological need thwarting through which they might impair nurses’ occupational wellbeing and turnover intentions.

Nurses’ memories of self-PMIEs were associated with lower work engagement, higher burnout, and more turnover intentions compared to both memories of other-PMIEs and memories of SMTs. Furthermore, memories of other-PMIEs were also associated with a similar decrease in nurses’ occupational wellbeing and, respectively, increase in turnover intentions compared to SMTs. These results support previous findings which showed that self-PMIEs have more negative effects on psychological health and functioning compared to other-PMIEs [5,13,15,16], in contrast to research showing similar effects for these two types of PMIEs [14].

However, these events thwarted basic psychological needs differently. While autonomy satisfaction was lower in memories of both self- and other-PMIEs compared to the control group, it was most thwarted for memories of self-PMIEs, which significantly mediated the associations with burnout, work engagement, and turnover intentions. Competence satisfaction, on the other hand, was highest in memories of other-PMIEs and lowest in memories of self-PMIEs and SMTs. Finally, relatedness satisfaction was lowest in memories of other-PMIEs compared to memories of SMTs and memories of self-PMIEs, but lower for self-PMIEs than for SMTs. This implies that autonomy thwarting could be the main mechanism through which exposure to self-PMIEs may affect long-term psycho-social functioning and health in nurses, while relatedness thwarting could play this role for exposure to other-PMIEs. Finally, nurses feeling ineffective and inefficacious following exposure to self-PMIEs is comparable to the deficits in competence satisfaction occurring after they committed an SMT at their workplace. From an intervention standpoint, this result would indicate that strategies such as the ones used for overcoming medical errors could be efficient in addressing competence thwarting following exposure to self-PMIEs.

Nurses’ experience of medical errors is arguably more complex compared to other healthcare providers due to their increased contact with patients, which often puts them in problematic situations after a medical error occurs [99]. Results of a systematic review suggest that disclosing medical errors to patients and family members enabled nurses to feel relief and closure, helping them to emotionally reconcile the event by taking the morally responsible action [100]. Organizational formal support and informal support from colleagues helped them restore personal integrity and implement constructive changes [101]. Future studies should investigate whether these two strategies could help restore nurses’ sense of competence following self-PMIEs, although disclosing such events as medical errors to patients would mean taking full responsibility for them, which could be problematic, because of the autonomy thwarting associated with self-PMIEs. Considering that they perpetrated those events under perceived external coercion, future studies should test disclosure to patients as a joint endeavor of the medical staff to alleviate nurses’ distress.

For the impact of the deficits in autonomy satisfaction associated with both memories of self- and other-PMIEs on our outcomes, we tested perceived supervisor support as a protective factor. Our results show that it operated as a protective factor against a decrease in work engagement for other-PMIEs, and against increases in turnover intentions for self- and other-PMIEs, without contributing to lowering burnout. Having a higher general level of perceived supervisor support could have helped nurses understand that the autonomy thwarting experienced during the self- and other-PMIEs (and associated with their memories of them) are not representative for their workplace and relationship with their supervisors outside of the crisis created by the COVID-19 pandemic. As such, in line with previous research, higher perceived supervisor support lowered the impact of autonomy thwarting on their turnover intentions [30,32]. This implies that interventions focused on increasing perceived supervisor support could help prevent and, possibly, restore the increase in nurses’ turnover intentions, attributable to exposure to self- and other-PMIEs during the COVID-19 pandemic. This also suggests that increasing perceived supervisor support could prevent the integration of these memories in nurses’ autobiographical knowledge.

On the other hand, perceived supervisor support did not protect against autonomy thwarting for any of the three groups. One possible explanation could be that burnout is considered a more complex syndrome, which severely affects occupational health, with some arguing it should be included as a distinct mental disorder in the current diagnostic system [102]. In contrast, work engagement is a positive, affective-motivational state of fulfillment, characterized by vigor, dedication, and absorption [46], without pathological elements [103]. Higher levels of self-disclosure can have therapeutic properties, as people usually act on their tendency to share their emotional experiences with others, whereas perceived supervisor support is more descriptive of work relationships. This might explain why higher self-disclosure was a significant moderator in our model for burnout, but not for perceived supervisor support.

Unlike perceived supervisor support, which is a result of previous experiences, to a certain extent, self-disclosure is a process of communication in which one naturally engages intentionally, with the purpose of sharing information about themselves and meaningful life events [34]. Previous studies suggest that self-disclosure leads to decreased loneliness [104,105], aiding people to perceive their contexts as empathic, helpful, and affirmative [106]. Thus, in opposition to perceived supervisor support, which describes a previous mode of relating, self-disclosure could have helped nurses experience social support *after* being exposed to PMIEs, and thus exert a reparatory influence. Our results on self-disclosure are in line with past research, which showed that recovery from moral injury was associated with seeking out social support [107] and reconnection activities [108]. This could also occur because sharing one’s experience could help them find redemptive meaning in these traumatic events, an ability essential for healing and moving past a PMIE [107,108,109]. In contrast, perceived lack of support following PMIEs led to sustained psychological distress and to the reinforcement of veterans’ sense of moral injury [110,111].

The stronger influence of self-disclosure for self-PMIEs can also be explained by past findings. Since self-PMIE exposure was associated with intense shame and guilt [15,16], disclosing personal information could open new perspectives about the self and the PMIE, fostering the construction of more affirmative narratives of the events [36]. Thus, self-disclosing emotions and information about a PMIE could help adaptive coping by moving from distrust and betrayal to bonding, trust, and empowerment [111].

Our research is not without limitations. Our research was cross-sectional, and we cannot derive any definitive conclusions regarding causality. Future studies should test our results longitudinally. Our sample was not representative of the population of Romanian nurses, although we aimed to collect data from nurses from various specialties. Furthermore, future studies should also explore the content of nurses’ memories of self- and other-PMIEs from the COVID-19 pandemic thematically. Although it would have enriched our research to have participants permit us to employ their data for this purpose, they did not agree, due to its delicate nature. Future studies should aim to achieve this purpose. Finally, we conducted three separate studies in 2022 (including the present study) in which we explored associations between Romanian nurses’ autobiographical episodic memories from the COVID-19 pandemic and several occupational health outcomes. For all three, we employed snowballing sampling. We first contacted participants who took part in previous studies on different topics and invited them to: (a) participate in the current study; and (b) to contact other potential participants from their personal networks who met the eligibility criteria. Since we extended this invitation for all three studies, we may have had people who took part in all three of them, which might have led to their becoming familiar with the purpose of our studies, since they were debriefed after each one. It should also be noted that their personal contacts may have shared socio-demographic characteristics with them, since they were more likely to contact friends/colleagues of similar age and background. This might have decreased the heterogeneity of our samples, but also the external validity of our findings. Future studies should test our results in different geo-cultural settings and on representative samples of nurses.

## 5. Conclusions

Our study experimentally assessed the differential associations between nurses’ autobiographical episodic memories of self- and other-PMIEs from the COVID-19 pandemic and burnout, work engagement, and turnover intentions, compared to a control group. We also explored whether differences in the three basic psychological needs (i.e., autonomy, competence, and relatedness) mediated this differential impact. In addition, we tested two potential protective factors: perceived supervisor support for autonomy satisfaction and self-disclosure for relatedness satisfaction. To the best of our knowledge, this is the first study which focuses on exploring these potential avenues for prevention and reparations following nurses’ exposure to self- and other-PMIEs during the COVID-19 pandemic. Our results suggest that both self- and other-PMIEs have unique associations with work engagement, burnout, and turnover intentions though different basic psychological need thwarting. As such, relatedness was more thwarted for memories of other-PMIEs, whereas competence and autonomy were more thwarted in memories of self-PMIEs. High perceived supervisor support can constitute a protective factor against the increase in turnover intentions associated to both types of PMIE memories, rendering the associations between autonomy thwarting and turnover intentions insignificant. It can also constitute a protective factor against the decrease in work engagement associated with memories of other-PMIEs, in a similar fashion. High self-disclosure can constitute a protective factor against the decrease in work engagement and, respectively, the increase in burnout, rendering the associations between relatedness thwarting and these two outcomes insignificant or less significant. All in all, our findings suggest that different strategies for moral repair should be employed to address the deleterious effects of the exposure of nurses to self- and other-PMIEs, highlighting the relevance of the nature of the outcome as well.

## Figures and Tables

**Table 1:** Participants’ characteristics.

	Experimental Condition						Total (*N* = 634)	
	Other-PMIE (*N* = 214)		Self-PMIE (*N* = 185)			SMT (*N* = 235)		
**Specialty**								
Dentistry	0	0%	1	0.54%	0	0%	1	0.15%
Emergency	23	10.75%	12	6.49%	20	8.51%	55	8.67%
Gastroenterology	6	2.8%	3	1.62%	8	3.4%	17	2.68%
Hematology	6	2.8%	7	3.78%	6	2.55%	19	3%
Intensive Care Units	20	9.35%	7	3.78%	16	6.81%	43	6.78%
Infectious Diseases	9	4.21%	8	4.32%	23	9.79%	40	6.31%
Internal Medicine	11	5.14%	11	5.95%	14	5.96%	36	5.68%
Chronic Internal Medicine	10	4.67%	13	7.03%	6	2.55%	29	4.57%
Neurology	16	7.48%	17	9.19%	20	8.51%	53	8.36%
Obstetrics Gynecology	7	3.27%	5	2.7%	12	5.11%	24	3.79%
Oncology	25	11.68%	26	14.05%	29	12.34%	80	12.62%
Palliation	24	11.21%	25	13.51%	34	14.47%	83	13.09%
Pneumology	12	5.61%	15	8.11%	10	4.26%	37	5.84%
Psychiatry	21	9.81%	10	5.41%	12	5.11%	43	6.78%
Radiology	2	0.94%	2	1.09%	2	0.85%	6	0.95%
Surgery	22	10.28%	23	12.43%	23	9.78%	68	10.73%
**Age**								
*M* ± *SD*	38.8 ± 8.75		37.3 ± 9.72		39.2 ± 8.49		38.5 ± 8.97	
Range	21–57		22–57		21–55		21–57	
**Sex**								
Female	191	89.25%	154	83.24%	193	82.13%	538	84.86%
Male	23	10.74%	31	16.76%	42	17.87%	96	15.14%
**Education**								
Post-secondary studies	186	86.92%	166	89.73%	218	92.76%	570	89.90%
Bachelor’s degree	20	9.35%	9	4.86%	12	5.11%	41	6.50%
Master’s degree	8	3.74%	10	5.41%	5	2.13%	23	3.60%
**Work experience (years)**								
*M* ± *SD*	13.6 ± 8.77		13 ± 10.4		13.9 ± 8.29		13.5 ± 9.11	
Range	1–36		1–38		1–35		1–38	

**Table 2:** Structural Model. Direct Relationships Testing H1–H4.

Paths	Path Coefficients	SE	T	95% CI	
				LL	UL
H1: S/SMT → A	−1.823	0.11	16.64	−1.999	−1.639
H1: O/S → A	0.836	0.109	7.669	0.658	1.016
H2: O/SMT → A	−0.986	0.106	9.306	−1.158	−0.811
H3: O/S → C	0.742	0.098	7.541	0.578	0.901
H3: O/SMT → C	0.744	0.087	8.544	0.601	0.883
H4: O/SMT → R	−1.536	0.111	13.875	−1.718	−1.355
H4: O/S → R	−0.677	0.113	5.997	−0.866	−0.493
H4: S/SMT → R	−0.858	0.112	7.682	−1.04	−0.671

Note: S/SMT = self-PMIEs compared to SMTs, O/S = other-PMIEs compared to self-PMIEs, O/SMT = other-PMIEs compared to SMTs, A = autonomy satisfaction, C = competence satisfaction, R = relatedness satisfaction.

**Table 3:** Mediation Analysis Results—H8.

Relationships	Total Effects					Direct Effects					Indirect Effects				
	Path Coeff.	SE	T	95%CI		Path Coeff.	SE	T	95%CI		Path Coeff.	SE	T	95%CI	
				LL	UL				LL	UL				LL	UL
H6 and H8: S/SMT → R → UWES → TI	1.563	0.252	6.211	1.162	1.994	−0.065	0.141	0.461	−0.292	0.172	0.083	0.03	2.733	0.044	0.149
H6 and H8: S/SMT → R → BRN → TI											0.038	0.019	1.985	0.015	0.08
H6 and H8: S/SMT → A → UWES → TI											0.168	0.058	2.877	0.089	0.286
H6 and H8: S/SMT → A → BRN → TI											0.043	0.032	1.33	0.003	0.11
H7 and H8: O/SMT → R → UWES → TI	1.203	0.210	5.721	0.866	1.561	−0.11	0.107	1.029	−0.283	0.067	0.149	0.05	2.964	0.081	0.251
H7 and H8: O/SMT → R → BRN → TI											0.067	0.032	2.074	0.027	0.138
H7 and H8: O/SMT → A → UWES → TI											0.091	0.033	2.75	0.048	0.159
H7 and H8: O/SMT → A → BRN → TI											0.023	0.018	1.313	0.002	0.061
H7 and H8: O/SMT → C → BRN → TI											−0.008	0.005	1.515	−0.019	−0.002
H7 and H8: O/SMT → C → UWES → TI											−0.012	0.007	1.655	−0.027	−0.003
H6 and H8: O/S → A → BRN → TI	−0.36	0.142	2.530	−0.588	−0.120	−0.045	0.107	0.42	−0.222	0.130	−0.02	0.015	1.308	−0.363	−0.047
H6 and H8: O/S → A → UWES → TI											−0.077	0.028	2.744	−0.149	−0.044
H6 and H8: O/S → C → BRN → TI											−0.008	0.005	1.523	0.008	0.036
H6 and H8: O/S → C → UWES → TI											−0.012	0.007	1.661	−0.025	0.012
H6 and H8: O/S → R → BRN → TI											0.03	0.015	1.991	−0.35	0.003
H6 and H8: O/S → R → UWES → TI											0.066	0.024	2.777	−0.019	−0.002

Note: S/SMT = self-PMIEs compared to SMTs, O/S = other-PMIEs compared to self-PMIEs, O/SMT = other-PMIEs compared to SMTs, A = autonomy satisfaction, C = competence satisfaction, R = relatedness satisfaction, BRN = burnout, UWES = work engagement, DDI = self-disclosure, TI = turnover intentions.

**Table 4:** Conditional Moderation Analyses.

Paths	Path Coefficient	SE	T	95%CI	
				LL	UL
O/SMT → A → BRN → TI conditional on PSS at −1 SD	0.016	0.008	1.912	0.005	0.033
O/SMT → A → BRN → TI conditional on PSS at Mean	0.013	0.006	1.984	0.005	0.027
O/SMT → A → BRN → TI conditional on PSS at +1 SD	0.01	0.007	1.345	0.001	0.026
O/SMT → A → UWES → TI conditional on PSS at −1 SD	0.043	0.014	3.022	0.024	0.073
O/SMT → A → UWES → TI conditional on PSS at Mean	0.024	0.009	2.595	0.012	0.044
O/SMT → A → UWES → TI conditional on PSS at +1 SD	0.005	0.01	0.532	−0.011	0.023
O/SMT → R → BRN → TI conditional on DDI at −1 SD	0.033	0.014	2.313	0.015	0.062
O/SMT → R → BRN → TI conditional on DDI at Mean	0.018	0.01	1.774	0.006	0.041
O/SMT → R → BRN → TI conditional on DDI at +1 SD	0.004	0.013	0.292	−0.015	0.028
O/SMT → R → UWES → TI conditional on DDI at −1 SD	0.062	0.02	3.136	0.035	0.102
O/SMT → R → UWES → TI conditional on DDI at Mean	0.025	0.013	1.864	0.006	0.051
O/SMT → R → UWES → TI conditional on DDI at +1 SD	−0.012	0.02	0.613	−0.048	0.017
S/SMT → A → BRN → TI conditional on PSS at −1 SD	0.029	0.015	1.94	0.01	0.06
S/SMT → A → BRN → TI conditional on PSS at Mean	0.024	0.012	2.006	0.009	0.049
S/SMT → A → BRN → TI conditional on PSS at +1 SD	0.018	0.014	1.352	0.002	0.048
S/SMT → A → UWES → TI conditional on PSS at −1 SD	0.08	0.025	3.15	0.045	0.13
S/SMT → A → UWES → TI conditional on PSS at Mean	0.045	0.017	2.641	0.022	0.08
S/SMT → A → UWES → TI conditional on PSS at +1 SD	0.01	0.019	0.532	−0.02	0.042
S/SMT → R → BRN → TI conditional on DDI at −1 SD	0.018	0.008	2.212	0.008	0.036
S/SMT → R → BRN → TI conditional on DDI at Mean	0.01	0.006	1.737	0.003	0.023
S/SMT → R → BRN → TI conditional on DDI at +1 SD	0.002	0.007	0.291	−0.008	0.016
S/SMT → R → UWES → TI conditional on DDI at −1 SD	0.035	0.012	2.899	0.019	0.06
S/SMT → R → UWES → TI conditional on DDI at Mean	0.014	0.008	1.827	0.004	0.029
S/SMT → R → UWES → TI conditional on DDI at +1 SD	−0.007	0.011	0.605	−0.027	0.009
O/S → A → BRN → TI conditional on PSS at −1 SD	−0.013	0.007	1.871	−0.029	−0.004
O/S → A → BRN → TI conditional on PSS at Mean	−0.011	0.006	1.922	−0.023	−0.004
O/S → A → BRN → TI conditional on PSS at +1 SD	−0.008	0.006	1.319	−0.023	−0.001
O/S → A → UWES → TI conditional on PSS at −1 SD	−0.036	0.012	2.946	−0.063	−0.02
O/S → A → UWES → TI conditional on PSS at Mean	−0.021	0.008	2.481	−0.038	−0.01
O/S → A → UWES → TI conditional on PSS at +1 SD	−0.005	0.009	0.525	−0.02	0.009
O/S → R → BRN → TI conditional on DDI at −1 SD	0.015	0.007	2.181	0.006	0.029
O/S → R → BRN → TI conditional on DDI at Mean	0.008	0.005	1.686	0.003	0.019
O/S → R → BRN → TI conditional on DDI at +1 SD	0.002	0.006	0.287	−0.006	0.013
O/S → R → UWES → TI conditional on DDI at −1 SD	0.027	0.01	2.879	0.015	0.048
O/S → R → UWES → TI conditional on DDI at Mean	0.011	0.006	1.765	0.003	0.024
O/S → R → UWES → TI conditional on DDI at +1 SD	−0.005	0.009	0.607	−0.021	0.007

Note: S/SMT = self-PMIEs compared to SMTs, O/S = other-PMIEs compared to self-PMIEs, O/SMT = other-PMIEs compared to SMTs, A = autonomy satisfaction, C = competence satisfaction, R = relatedness satisfaction, BRN = burnout, UWES = work engagement, DDI = self-disclosure, TI = turnover intentions.

## Data Availability

The data presented in this study are available on request from the corresponding author. The data are not publicly available due to ethical constraints created by the sensitive topic investigated, which precludes us from sharing the content of the memories of moral transgressions recalled by participants, as well as information based on which participants could be identified.

## Appendix A

### Experimental Procedure

First, all participants read definitions and examples for the roles of “moral victims” and “moral transgressors”. Moral transgressors were defined as “individuals whose intentions and actions bring about harmful events” and moral victims as “individuals who experience feelings and emotions brought about by the moral transgressor’s actions” [9]. They were told that the same person can be a moral transgressor or a moral victim at different times or at the same time. The examples of moral transgressors and victims were the ones used by Gherman et al. [12], fitted for the nurses’ work environment and depicting severe moral transgressions (i.e., having had harmful effects for patients) [20,22]. The example for the role of moral transgressor was:

“*Laura is a nurse at a hospital in Romania. One morning during the 4th wave of the COVID-19 pandemic, she woke up feeling sick, and tested herself for COVID-19 at home with three rapid tests. Although all three tests were positive, she went to work anyway, because she had the opportunity to work an extra shift and make more money. Patients and colleagues contracted the infection from her and several of them are still in the ICU, with reserved prognoses. Laura felt terribly guilty and ashamed about the consequences of her action*”.

The example for the role of moral victim was:

“*Laura is a nurse at a hospital in Romania. During the 4th wave of the COVID-19 pandemic, she unknowingly cared for a patient who was infected with COVID-19. The patient knew about the infection, but lied about it, taking advantage of the fact that patients were not tested prior to being committed. Laura, along with several other patients and colleagues, contracted COVID-19 from the patient, and she is now in the ICU, with a reserved prognosis. Laura felt betrayed and angered about the consequences of the patient’s action*”.

Next, all participants read definitions and examples for self-PMIEs, other-PMIEs, and SMTs. All examples depicted severe moral transgressions, with severity operationalized as the magnitude of the harmful effects on patients [18] and were devised according to Brüggemann et al. [18] and Gherman et al. [12].

Self-PMIEs were defined as: “events or action during which you felt as both a moral victim and a moral transgressor, when you did something that you felt was morally wrong not because you wanted to, but because you felt as if you did not have a choice” [9].

The example for self-PMIEs was: “Laura is a nurse at a hospital in Romania. During the 4th wave of the COVID-19 pandemic, the beds in the ICU were all occupied, and she had to care for several patients with COVID-19 in the ER. Four patients were rapidly deteriorating, all of them badly needing access to ventilators. None were available, and Laura had to decide to start manual ventilation on one of them. The physicians on call were not answering, and patients’ oxygen saturations were dropping quickly. In the spur of the moment, she started the procedure on the youngest patient, a 12-year-old child. Until other nurses could join her, the oldest patient of the four died. Laura felt incredibly guilty for not having saved his life”.

Other-PMIEs were defined as: “events or action during which you felt as both a moral victim and a moral transgressor, when you witnessed something that you felt was morally wrong and failed to act or speak out not because you wanted to, but because you felt as if you did not have a choice” [9].

The example for others-PMIEs was: “Laura is a nurse at a hospital in Romania. During the 4th wave of the COVID-19 pandemic, the beds in the ICU were all occupied, and she had to care for several patients with COVID-19 in the ER. Four patients were rapidly deteriorating, all of them badly needing access to ventilators. None were available, and the physician on call had to decide to start manual ventilation on two of them. He told Laura to start the procedure on the youngest patient, a 12-year-old child, while he proceeded to do the same on a 26-year-old female. Until other medical staff could join them, the oldest patient of the four died. Laura felt incredibly guilty for not having saved his life, since, in her opinion, his condition was the most critical of the four, but felt that she could not have disobeyed the doctor’s order”.

SMTs were defined as: “events or action during which you felt as a moral transgressor, when you did something that you felt was morally wrong in the absence of any external pressures to do so” [9,12].

The example for SMTs was the same as the one for the role of moral transgressor, previously used by Gherman et al. [12]: “Laura is a nurse at a hospital in Romania. One morning during the 4th wave of the COVID-19 pandemic, she woke up feeling sick, and tested herself for COVID-19 at home with three rapid tests. Although all three tests were positive, she went to work anyway, because she had the opportunity to work an extra shift and make more money. Patients and colleagues contracted the infection from her and several of them are still in the ICU, with reserved prognoses. Laura felt terribly guilty and ashamed about the consequences of her action”.

All examples depicted work-related incidents to prepare participants for the recall tasks.

Next, participants in the self-PMIE condition received the following instruction, adapted from previous research [12,13,52,63,64,65,66,67,68,69]: “Please describe a personal memory of a specific event related to your work during the COVID-19 pandemic which you consider a self-PMIE, as defined and exemplified above. Select a memory significant to you which is at least six-months-old, and which often comes to your mind. This memory should be of the most morally wrong thing you have done during the pandemic with harmful consequences on a patient, under environmental constraints. Describe in a general fashion what happened, where it happened, who you were with (if anyone), and how you and other people reacted. Please remember we are not interested in the identities of anybody involved, so feel free to use phrases such as ‘a colleague’, ‘a boss’, ‘a patient’, and other generic denominators. What is important to us is for you to remember specific details, not for us to know them. Describe your role and what have been the consequences of your reaction or of your actions during this event. Please provide enough details so that we can fully understand what happened, as if you were telling a story to someone. We would also like to assure you that the content of your memories will not be shared with anybody outside of the two first authors and it will not be used in our analyses”.

Participants in the other-PMIE condition received the same instruction, with the first two sentences modified as such: “Please describe a personal memory of a specific event related to your work during the COVID-19 pandemic which you consider an other-PMIE, as defined and exemplified above. Select a memory significant to you which is at least six-months-old, and which often comes to your mind. This memory should be of the most morally wrong thing you have witnessed during the pandemic with harmful consequences on a patient, against which you wanted to speak out or take action, but you felt you could not”.

Finally, participants in the SMT condition also received the same instruction, with the first two sentences modified as such: “Please describe a personal memory of a specific event related to your work during the COVID-19 pandemic which you consider a severe moral transgression, as defined and exemplified above. Select a memory significant to you which is at least six-months-old, and which often comes to your mind. This memory should be of the most morally wrong thing you have done during the pandemic with harmful consequences on a patient, without any external pressure to do so”.

## Appendix B

### Manipulation Checks Results

To check for differences in perceived moral severity of the events recalled across the three experimental conditions, we conducted a one-way ANOVA, which showed no significant differences between the three groups: *F*_(6, 631)_ = 0.08, *p* = 0.923, *η*^2^ = 0 (Table A1).

To assess differences between the three experimental groups according to the perceived role in the event (i.e., witness, moral victim, moral perpetrator), we ran a mixed repeated measures ANOVA, with the experimental group as the between-subjects factor, and the perceived role as the within-subjects factor. Mauchly’s test of sphericity showed the violation of the assumption of sphericity, *χ*^2^_(2)_ = 0.906, *p* < 0.001, so we used a Greenhouse–Geisser correction for within-subjects effects. The results of the three-way mixed ANOVA showed significant main effects for type of role (*F*_(1.83, 1153.54)_ = 418, *p* < 0.001, *η_p_*^2^ = 0.399), experimental condition (*F*_(2, 631)_ = 465, *p* < 0.001, *η_p_*^2^ = 0.596), and the interaction between them (*F*_(3.66, 1153.54)_ = 587, *p* < 0.001, *η_p_*^2^ = 0.651). Post-hoc tests with Tukey corrections showed that, for the witness role, there were no significant differences between the SMT (*M* = 1.96, *SD* = 0.83) and the self-PMIE (*M* = 2.16, *SD* = 1.01) groups (*t*_(631)_ = 2.18, *p* = 0.417), and people recalling other-PMIEs (*M* = 5.91, *SD* = 0.99) perceived themselves as moral transgression witnesses significantly more than the ones recalling self-PMIEs (*t*_(631)_ = 41.27, *p* < 0.001) and SMTs (*t*_(631)_ = −41.58, *p* < 0.001). For the perpetrator role, there were no significant differences between the SMT (*M* = 5.85, *SD* = 1) and the self-PMIE (*M* = 5.85, *SD* = 1.15) groups (*t*_(631)_ = 0.02, *p* = 1), and people recalling other-PMIEs (*M* = 4.08, *SD* = 2.01) perceived themselves as moral transgressors significantly less than the ones recalling self-PMIEs (*t*_(631)_ = −11.99, *p* < 0.001) and SMTs (*t*_(631)_ = 12.76, *p* < 0.001). For the victim role, there were no significant differences between the other-PMIE (*M* = 5.87, *SD* = 1.1) and the self-PMIE (*M* = 5.83, *SD* = 1.07) groups (*t*_(631)_ = 0.39, *p* = 1), and people recalling SMTs (*M* = 2.11, *SD* = 1.03) perceived themselves as moral victims significantly less than the ones recalling self-PMIEs (*t*_(631)_ = −35.4, *p* < 0.001) and other-PMIEs (*t*_(631)_ = −37.24, *p* < 0.001). People recalling SMTs felt less like moral witnesses than moral perpetrators (*t*_(631)_ = −31.86, *p* < 0.001), and more like moral perpetrators than moral victims (*t*_(631)_ = 31.05, *p* < 0.001), with no significant differences between the roles of moral witness and moral victim (*t*_(631)_ = 0.55, *p* = 1). People recalling other-PMIEs felt more like moral witnesses than moral perpetrators (*t*_(631)_ = 15.06, *p* < 0.001), and less like moral perpetrators than moral victims (*t*_(631)_ = −14.14, *p* < 0.001), with no significant differences between the roles of moral witness and moral victim (*t*_(631)_ = 0.44, *p* = 1). People recalling self-PMIEs felt less like moral witnesses than moral perpetrators (*t*_(631)_ = −29.82, *p* < 0.001), and less like moral witnesses than moral victims (*t*_(631)_ = −37.33, *p* < 0.001), with no significant differences between the roles of moral perpetrator and moral victim (*t*_(631)_ = 0.16, *p* = 1).

**Table A1:** Differences between the three experimental groups according to perceived moral severity, age, work experience, perceived supervisor support, self-disclosure, sex, education.

	Self-PMIE	Other-PMIE	SMT				
	*M* ± *SD*	*M* ± *SD*	*M* ± *SD*	*F*	*df*	*p*	*η* ^2^
Moral severity	6.06 ± 0.8	6.07 ± 0.82	6.04 ± 0.76	0.08	2, 631	0.923	0
Age	39.2 ± 8.49	38.8 ± 8.75	37.3 ± 9.72	2.66	2, 631	0.07	0.008
Work experience	13.9 ± 8.29	13.6 ± 8.78	13 ± 10.4	0.43	2, 631	0.65	0.001
PSS	21.1 ± 5.37	21.4 ± 5.28	20.9 ± 7.28	0.31	2, 631	0.735	0.001
Self-Disclosure	35.3 ± 10.7	35.2 ± 10.3	36.1 ± 11	0.39	2, 632	0.674	0.001
	Observed frequencies			χ^2^	*df*	*p*	*n*
Sex (Male/Female)	31/154	23/191	42/193	4.95	2	0.084	634
Education (PS/B/M)	166/9/10	186/20/8	218/12/5	7.65	4	0.105	634

Note: SMT = severe moral transgressions; self-PMIEs = potentially morally injurious events perpetrated by the self; other-PMIEs = potentially morally injurious events witnessed by the self; PS = Post-secondary Studies; B = Bachelor’s Degree; M = Master’s Degree; PSS = perceived supervisor support.

## Appendix C

**Table A2:** Socio-demographic differences in perceived supervisor support, self-disclosure, work engagement, emotional exhaustion, depersonalization, personal accomplishment, burnout, and turnover intentions.

Characteristics	*N*	*M* ± *SD*	*t*/*F*	Cohen’s *d*/η^2^	Post-Hoc Tests ^a^
Perceived Supervisor Support					
**Sex**			*t*(632) = −0.23, *p* = 0.818	−0.03	
Male	96	21 ± 6.53			
Female	538	21.2 ± 5.86			
**Work experience (years)**			*t*(632) = −1.95, *p* = 0.052	−0.16	-
0.5–10	298	20.6 ± 6.1			
11–38	336	21.6 ± 5.8			
**Age (years)**			*F* (2, 631) = 1.19, *p* = 0.304	0	
21–30	144	20.5 ± 6.27			
31–40	232	21.1 ± 5.86			
41–57	158	21.5 ± 5.86			
**Education**			*F*(2, 631) = 2.18, *p* = 0.113	0.01	-
PS	570	21 ± 5.95			
B	41	23 ± 5.89			
M	23	20.8 ± 6.01			
**Self-Disclosure**					
**Sex**			*t*(632) = −0.34, *p* = 0.732	−0.04	
Male	96	35.1 ± 10.89			
Female	538	35.5 ± 10.58			
**Work experience (years)**			*t*(632) = −3.26, *p* = 0.001	−0.26	-
0.5–10	298	34 ± 10.61			
11–38	336	36.8 ± 10.49			
**Age (years)**			*F* (2, 631) = 4.48, *p* = 0.012	0.01	21–30 < 41–57 *
21–30	144	33.8 ± 10.8			
31–40	232	34.9 ± 10			
41–57	158	36.9 ± 10.9			
**Education**			*F*(2, 631) = 1.07, *p* = 0.345	0	-
PS	570	35.6 ± 10.7			
B	41	34.9 ± 11.5			
M	23	32.4 ± 6.79			
**Work Engagement**					
**Sex**			*t*(632) = −0.43, *p* = 0.670	−0.05	
Male	96	30.4 ± 9.6			
Female	538	30.8 ± 8.41			
**Work experience (years)**			*t*(632) = −3.75, *p* < 0.001	−0.3	-
0.5–10	298	29.4 ± 8.53			
11–38	336	31.9 ± 8.48			
**Age (years)**			*F* (2, 631) = 10.9, *p* < 0.001	0.03	21–30 < 31–40 ***, 21–30 < 41–57 ***
21–30	144	27.9 ± 8.49			
31–40	232	31.4 ± 8.12			
41–57	158	31.7 ± 8.74			
**Education**			*F*(2, 631) = 4.54, *p* = 0.011	0.01	PS > M *, B > M **
PS	570	30.8 ± 8.71			
B	41	32.9 ± 7.69			
M	23	26.2 ± 5.08			
**Emotional Exhaustion**					
**Sex**			*t*(632) = 0.14, *p* = 0.889	0.02	-
Male	96	29 ± 12.11			
Female	538	28.9 ± 11.52			
**Work experience (years)**			*t*(632) = 2.66, *p* = 0.008	0.21	-
0.5–10	298	30.2 ± 11.64			
11–38	336	27.7 ± 11.46			
**Age (years)**			*F*(2, 631) = 8.63, *p* < 0.001	0.03	21–30 > 31–40 *, 21–30 > 41–57 ***
21–30	144	32 ± 11.2			
31–40	232	29 ± 11.5			
41–57	158	27.1 ± 11.6			
**Education**			*F*(2, 631) = 1.5, *p* = 0.223	0.01	-
PS	570	28.6 ± 11.8			
B	41	30.3 ± 9.14			
M	23	32.4 ± 9.1			
**Depersonalization**					
**Sex**			^b^*t*(120) = −0.2, *p* = 0.845	−0.02	-
Male	96	15.4 ± 7.45			
Female	538	15.6 ± 6.21			
**Work experience (years)**			*t*(632) = 2.89, *p* = 0.004	0.23	-
0.5–10	298	16.4 ± 6.43			
11–38	336	14.9 ± 6.32			
**Age (years)**			*F*(2, 631) = 1.63, *p* = 0.197	0.05	
21–30	144	16.4 ± 6.54			
31–40	232	15.4 ± 6.36			
41–57	158	15.3 ± 6.36			
**Education**			*F*(2, 631) = 3.86, *p* = 0.022	0.01	PS < B *, PS < M *
PS	570	15.5 ± 6.41			
B	41	15.1 ± 5.88			
M	23	19.2 ± 6.34			
**Personal Accomplishment**					
**Sex**			*t*(632) = 0.28, *p* = 0.779	0.03	-
Male	96	26.5 ± 11.6			
Female	538	26.1 ± 10.78			
**Work experience (years)**			*t*(632) = 3.39, *p* < 0.001	0.27	-
0.5–10	298	27.7 ± 10.79			
11–38	336	24.8 ± 10.82			
**Age (years)**			*F*(2, 631) = 5.21, *p* = 0.006	0.02	21–30 > 41–57 **
21–30	144	28.4 ± 11.3			
31–40	232	26.3 ± 10.3			
41–57	158	24.8 ± 11			
**Education**			*F*(2, 631) = 1.45, *p* = 0.236	0.01	-
PS	570	26 ± 11.1			
B	41	26.7 ± 9.79			
M	23	29.9 ± 8.19			
**Burnout**					
**Sex**			*t*(632) = 0.14, *p* = 0.886	0.02	-
Male	96	70.9 ± 24.21			
Female	538	70.6 ± 22.45			
**Work experience (years)**			*t*(632) = 3.82, *p* < 0.001	0.3	-
0.5–10	298	74.3 ± 22.93			
11–38	336	67.4 ± 22.04			
**Age (years)**			*F*(2, 631) = 8.7, *p* < 0.001	0.03	21–30 > 31–40 *, 21–30 > 41–57 ***
21–30	144	76.8 ± 23.5			
31–40	232	70.7 ± 21.7			
41–57	158	67.1 ± 22.4			
**Education**			*F*(2, 631) = 2.87, *p* = 0.057	0.01	-
PS	570	70.1 ± 23			
B	41	72 ± 19.9			
M	23	81.5 ± 16.5			
**Turnover Intentions**					
**Sex**			*t*(632) = 1.3, *p* = 0.194	0.14	-
Male	96	13.7 ± 3.55			
Female	538	13.2 ± 3.53			
**Work experience (years)**			*t*(632) = 1.43, *p* = 0.152	0.11	-
0.5–10	298	13.5 ± 3.49			
11–38	336	13.1 ± 3.56			
**Age (years)**			*F*(2, 631) = 3.7, *p* = 0.025	0.01	21–30 > 31–40 *, 21–30 > 41–57 *
21–30	144	14 ± 3.47			
31–40	232	13 ± 3.28			
41–57	158	13.1 ± 3.74			
**Education**			*F*(2, 631) = 3.64, *p* = 0.027	0.01	B < M *
PS	570	13.3 ± 3.55			
B	41	12 ± 3.32			
M	23	14.3 ± 2.91			

Note: ^a^ Only significant post-hoc tests are summarily presented, ^b^ Levene’s test was significant (*p* < 0.05), suggesting a violation of the assumption of equal variances. Therefore, Welch’s *t*-test was reported. *** *p* < 0.001, ** *p* < 0.01, * *p* < 0.05. PS = Post-Secondary Studies; B = Bachelor’s Degree; M = Master’s Degree.

**Table A3:** Correlations between basic psychological need satisfaction in autobiographical memories, self-disclosure, perceived supervisor support, wellbeing, and turnover intentions. Skewness and kurtosis.

		Skewness	Kurtosis	1		2		3		4		5		6		7	
**1**	**Burnout**	−0.03	−0.84	—													
**2**	**Work engagement**	0.03	−0.72	−0.61	***	—											
**3**	**Turnover intentions**	−0.09	−0.23	0.44	***	−0.45	***	—									
**4**	**Perceived supervisor support**	0.33	−0.04	−0.15	***	0.11	**	−0.51	***	—							
**5**	**Self-disclosure**	0.11	−0.56	−0.46	***	0.06		−0.13	**	0.11	**	—					
**6**	**Autonomy**	0.12	−0.57	−0.42	***	0.41	***	−0.3	***	−0.02		0.08	*	—			
**7**	**Competence**	0.29	−0.41	−0.11	**	0.07		−0.09	*	0.16	***	0.17	***	−0.01		—	
**8**	**Relatedness**	0.11	−0.46	−0.25	***	0.24	***	−0.2	***	0.06		−0.01		0.22	***	−0.18	***

Note: * *p* < 0.05, ** *p* < 0.01, *** *p* < 0.001.

## Appendix D

**Table A4:** Stage 1—Outer loadings, Reliability, Convergent Validity.

LOCs	Items	Outer Loadings	Alpha	rho_A	rho_C	AVE
A	A1	0.966	0.924	0.927	0.963	0.929
	A2	0.961				
C	C1	0.935	0.862	0.864	0.936	0.879
	C2	0.94				
R	R1	0.958	0.911	0.911	0.957	0.918
	R2	0.958				
Vigor	UWE1	0.957	0.958	0.965	0.972	0.921
	UWE2	0.962				
	UWE3	0.96				
Absorption	UWE4	0.96	0.96	0.964	0.974	0.926
	UWE5	0.965				
	UWE6	0.962				
Dedication	UWE7	0.962	0.957	0.957	0.972	0.921
	UWE8	0.958				
	UWE9	0.959				
EE	EE1	0.911	0.985	0.985	0.987	0.894
	EE2	0.952				
	EE3	0.953				
	EE4	0.95				
	EE5	0.948				
	EE6	0.949				
	EE7	0.945				
	EE8	0.951				
	EE9	0.953				
DP	DP1	0.959	0.978	0.979	0.982	0.918
	DP2	0.956				
	DP3	0.96				
	DP4	0.955				
	DP5	0.959				
PA	PA1	0.954	0.984	0.984	0.987	0.913
	PA2	0.954				
	PA3	0.956				
	PA4	0.959				
	PA5	0.958				
	PA6	0.956				
	PA7	0.952				
PSS	PSS1	0.856	0.933	0.939	0.947	0.75
	PSS2	0.861				
	PSS3	0.867				
	PSS4	0.87				
	PSS5	0.876				
	PSS6	0.866				
DDI	S1	0.856	0.966	0.967	0.97	0.73
	S2	0.828				
	S3	0.842				
	S4	0.852				
	S5	0.862				
	S6	0.839				
	S7	0.874				
	S8	0.861				
	S9	0.852				
	S10	0.857				
	S11	0.86				
	S12	0.867				
TI	TI1	0.949	0.944	0.944	0.964	0.899
	TI2	0.945				
	TI3	0.95				

Note. TI = Turnover Intentions, A = Autonomy satisfaction, C = Competence satisfaction, R = Relatedness satisfaction, DDI = Self-disclosure, PSS = Perceived Supervisor Support, PA = Personal Accomplishment, DP = Depersonalization, EE = Emotional Exhaustion.

**Table A5:** Stage 1—Discriminant Validity according to HTMT Values and the Fornell–Larcker Criterion.

		1	2	3	4	5	6	7	8	9	10	11	12
1	A	***0.964***	0.32	0.016	0.357	0.334	0.38	0.319	0.034	0.238	0.088	0.318	0.364
2	Absorption	0.302	***0.962***	0.077	0.346	0.434	0.417	0.359	0.126	0.16	0.073	0.365	0.401
3	C	−0.007	0.071	***0.938***	0.112	0.067	0.063	0.113	0.175	0.205	0.181	0.097	0.038
4	DP	−0.34	−0.337	−0.102	***0.958***	0.352	0.431	0.387	0.119	0.177	0.243	0.316	0.365
5	Dedication	0.314	0.417	0.06	−0.341	***0.96***	0.405	0.376	0.06	0.203	0.041	0.353	0.421
6	EE	−0.364	−0.407	−0.058	0.424	−0.394	***0.946***	0.429	0.103	0.193	0.418	0.366	0.399
7	PA	−0.304	−0.35	−0.104	0.381	−0.365	0.424	***0.955***	0.144	0.24	0.387	0.371	0.38
8	PAS	−0.016	0.122	0.154	−0.115	0.058	−0.101	−0.139	***0.866***	0.067	0.12	0.543	0.087
9	R	0.218	0.15	−0.182	−0.167	0.189	−0.183	−0.227	0.064	***0.958***	0.024	0.211	0.223
10	SD	0.084	0.072	0.166	−0.238	0.04	−0.409	−0.379	0.114	−0.006	***0.854***	0.133	0.028
11	TI	−0.298	−0.349	−0.087	0.304	−0.336	0.353	0.358	−0.51	−0.196	−0.127	***0.948***	0.395
12	Vigor	0.344	0.387	0.035	−0.355	0.405	−0.391	−0.371	0.083	0.209	0.019	−0.377	***0.96***

Note. Diagonal and bolded, italicized are the square roots of the AVE. Below the diagonal elements are correlations between constructs’ values. Above the diagonal elements are the HTMT values. TI = Turnover Intentions, A = Autonomy satisfaction, C = Competence satisfaction, R = Relatedness satisfaction, DDI = Self-disclosure, PSS = Perceived Supervisor Support, PA = Personal Accomplishment, DP = Depersonalization, EE = Emotional Exhaustion.

**Table A6:** Stage 2—Higher Order Constructs Validation: Multicollinearity Analysis, Outer Weights, and Outer Loadings.

HOCs	LOCs	Items	VIF	Outer Weights		Outer Loadings	
	A	A1	3.793	0.54	***	0.967	***
		A2	3.793	0.497	***	0.961	***
	C	C1	2.352	0.515	***	0.933	***
		C2	2.352	0.552	***	0.942	***
	R	R1	3.32	0.523	***	0.958	***
		R2	3.32	0.521	***	0.958	***
UWES	Vigor	-	1.285	0.507	***	0.816	***
	Absorption	-	1.3	0.388	***	0.747	***
	Dedication	-	1.322	0.391	***	0.758	***
BRN	EE	-	1.352	0.509	***	0.831	***
	DP	-	1.297	0.276	***	0.676	***
	PA	-	1.297	0.485	***	0.806	***
	PSS	PSS1	2.834	0.162	***	0.855	***
		PSS2	2.61	0.232	***	0.864	***
		PSS3	2.942	0.173	***	0.866	***
		PSS4	2.826	0.211	***	0.869	***
		PSS5	3.008	0.189	***	0.874	***
		PSS6	2.834	0.188	***	0.865	***
	DDI	S1	3.204	0.096	***	0.856	***
		S2	2.842	0.079	***	0.828	***
		S3	3.018	0.09	***	0.842	***
		S4	3.116	0.1	***	0.852	***
		S5	3.297	0.102	***	0.862	***
		S6	2.893	0.096	***	0.839	***
		S7	3.553	0.105	***	0.874	***
		S8	3.299	0.098	***	0.861	***
		S9	3.096	0.1	***	0.852	***
		S10	3.216	0.101	***	0.857	***
		S11	3.23	0.106	***	0.86	***
		S12	3.452	0.096	***	0.867	***
	TI	TI1	4.494	0.36	***	0.949	***
		TI2	4.343	0.351	***	0.945	***
		TI3	4.768	0.344	***	0.95	***

Note. HOC = Higher-Order Constructs, LOC = Lower-Order Constructs, BRN = Burnout, UWES = Work Engagement, TI = Turnover Intentions, A = Autonomy satisfaction, C = Competence satisfaction, R = Relatedness satisfaction, DDI = Self-disclosure, PSS = Perceived Supervisor Support, PA = Personal Accomplishment, DP = Depersonalization, EE = Emotional Exhaustion. *** *p* < 0.001.

**Table A7:** Explanatory and Predictive Power of the Model.

Predictors	Outcomes	R2	f2	Q2
O/S	TI	0.467	0.011	0.945
SMT/S			0.011	
O/SMT			0.012	
A			0.033	
C			0.011	
R			0.014	
BRN			0.03	
UWES			0.07	
PSS × A			0.012	
DDI × R			0.011	
PSS			0.383	
DDI			0.011	
O/S	BRN	0.586	0.146	0.568
SMT/S			0.45	
O/SMT			0.125	
A			0.023	
C			0.018	
R			0.026	
PSS × A			0.014	
DDI × R			0.015	
PSS			0.027	
DDI			0.414	
O/S	UWES	0.43	0.168	0.383
SMT/S			0.352	
O/SMT			0.083	
A			0.038	
C			0.033	
R			0.04	
PSS × A			0.041	
DDI × R			0.029	
PSS			0.039	
DDI			0.031	
O/S	A	0.308	0.088	0.433
O/SMT			0.139	
SMT/S			0.438	
O/S	C	0.112	0.088	0.629
O/SMT			0.126	
SMT/S			0	
O/S	R	0.24	0.054	0.356
O/SMT			0.313	
SMT/S			0.09	

Note. O/S = other-PMIEs compared to self-PMIEs, O/SMT = other-PMIEs compared to SMTs, SMT/S = SMTs compared to self-PMIEs, BRN = Burnout, UWES = Work Engagement, TI = Turnover Intentions, A = Autonomy satisfaction, C = Competence satisfaction, R = Relatedness satisfaction, DDI = Self-disclosure, PSS = Perceived Supervisor Support, PA = Personal Accomplishment, DP = Depersonalization, EE = Emotional Exhaustion.

**Table A8:** Predictive Power of the Model.

Endogenous Constructs	Indicators	Q^2^ Predict	PLS-SEM_RMSE	LM_RMSE
A	A1	0.32	1.099	1.105
	A2	0.247	1.254	1.265
C	C1	0.085	1.092	1.073
	C2	0.107	1.044	1.041
R	R1	0.217	1.248	1.27
	R2	0.215	1.201	1.219
UWES	Vigor	0.252	0.866	0.888
	Absorption	0.216	0.887	0.903
	Dedication	0.23	0.879	0.906
BRN	EE	0.405	0.772	0.787
	DP	0.254	0.865	0.881
	PA	0.363	0.799	0.805
TI	TI1	0.15	1.081	0.973
	TI2	0.144	1.212	1.093
	TI3	0.14	1.153	1.053

Note. BRN = Burnout, UWES = Work Engagement, TI = Turnover Intentions, A = Autonomy satisfaction, C = Competence satisfaction, R = Relatedness satisfaction, DDI = Self-disclosure, PSS = Perceived Supervisor Support, PA = Personal Accomplishment, DP = Depersonalization, EE = Emotional Exhaustion.

**Table A9:** Mediation Analyses—Other Results.

Paths	Total Indirect Effects					Direct Effects					Indirect Effects				
	Path Coeff	SE	T	95%CI		Path Coeff	SE	T	95%CI		Path Coeff	SE	T	95%CI	
				LL	UL				LL	UL				LL	UL
A → BRN → TI											−0.02	0.018	1.339	−0.06	−0
A → UWES → TI	−0.12	0.037	3.124	−0.18	−0.06	−0.235	0.113	2.084	−0.42	−0.05	−0.09	0.032	2.845	−0.16	−0.05
C → BRN → TI											−0.01	0.007	1.569	−0.02	−0
C → UWES → TI	−0.03	0.012	2.153	−0.05	−0.01	0.02	0.036	0.556	−0.04	0.078	−0.02	0.009	1.706	−0.04	−0
O/SMT → A → BRN											0.093	0.059	1.586	0.002	0.195
O/SMT → C → BRN											−0.03	0.016	1.902	−0.06	−0.01
O/SMT → R → BRN	0.332	0.11	3.009	0.155	0.524	0.494	0.054	9.092	0.401	0.579	0.27	0.092	2.92	0.122	0.426
O/SMT → A → TI											0.232	0.118	1.974	0.051	0.438
O/SMT → BRN → TI											0.123	0.043	2.833	0.056	0.199
O/SMT → C → TI											0.015	0.027	0.552	−0.03	0.059
O/SMT → R → TI											0.383	0.144	2.654	0.155	0.629
O/SMT → UWES → TI	1.203	0.21	5.721	0.866	1.561	−0.11	0.107	1.029	−0.28	0.067	0.139	0.038	3.672	0.085	0.211
O/SMT → A → UWES											−0.32	0.088	3.615	−0.48	−0.19
O/SMT → C → UWES											0.042	0.024	1.783	0.007	0.084
O/SMT → R → UWES	−0.8	0.155	5.13	−1.05	−0.55	−0.487	0.087	5.57	−0.63	−0.34	−0.52	0.125	4.148	−0.73	−0.32
PSS → BRN → TI											−0.01	0.007	1.838	−0.03	−0
PSS → UWES → TI	−0.02	0.014	1.335	−0.05	0.002	−0.522	0.054	9.706	−0.61	−0.43	−0.01	0.011	0.544	−0.03	0.012
PSS x A → BRN → TI											0.003	0.004	0.666	−0	0.012
PSS x A → UWES → TI	0.022	0.01	2.301	0.008	0.039	0.03	0.03	0.981	−0.02	0.081	0.019	0.008	2.364	0.008	0.036
R → BRN → TI											−0.04	0.021	2.115	−0.09	−0.02
R → UWES → TI	−0.14	0.038	3.695	−0.21	−0.09	−0.25	0.091	2.729	−0.4	−0.1	−0.1	0.032	3.028	−0.16	−0.05
DDI → BRN → TI											−0.09	0.029	2.934	−0.14	−0.04
DDI → UWES → TI	−0.08	0.034	2.435	−0.14	−0.03	0.061	0.056	1.095	−0.03	0.154	0.003	0.013	0.196	−0.02	0.025
DDI x R → BRN → TI											0.011	0.006	1.659	0.003	0.025
DDI x R → UWES → TI	0.038	0.013	2.871	0.018	0.062	0.07	0.031	2.255	0.019	0.121	0.027	0.01	2.622	0.013	0.048
S/SMT → A → BRN											0.172	0.107	1.611	−0	0.35
S/SMT → R → BRN	0.323	0.122	2.651	0.13	0.53	0.993	0.071	14.03	0.869	1.101	0.151	0.056	2.702	0.069	0.255
S/SMT → A → UWES											−0.59	0.151	3.885	−0.84	−0.35
S/SMT → R → UWES	−0.88	0.17	5.148	−1.17	−0.61	−1.19	0.098	12.18	−1.35	−1.03	−0.29	0.081	3.599	−0.44	−0.17
S/SMT → A → TI											0.429	0.208	2.066	0.091	0.776
S/SMT → BRN → TI											0.247	0.086	2.884	0.11	0.39
S/SMT → R → TI											0.214	0.085	2.524	0.088	0.369
S/SMT → UWES → TI	1.563	0.252	6.211	1.162	1.994	−0.065	0.141	0.461	−0.29	0.172	0.341	0.075	4.547	0.225	0.473
O/S → A → BRN											−0.08	0.05	1.577	−0.17	−0
O/S → C → BRN											−0.03	0.016	1.909	−0.06	−0.01
O/S → R → BRN	−0.49	0.084	5.81	−0.62	−0.34	−0.499	0.059	8.426	−0.59	−0.4	0.119	0.044	2.734	0.056	0.199
O/S → A → UWES											0.269	0.075	3.571	0.158	0.407
O/S → C → UWES											0.042	0.023	1.798	0.007	0.083
O/S → R → UWES	0.785	0.116	6.771	0.589	0.969	0.704	0.074	9.517	0.578	0.823	−0.23	0.062	3.697	−0.34	−0.14
O/S → A → TI											−0.2	0.095	2.062	−0.36	−0.05
O/S → BRN → TI											−0.12	0.046	2.731	−0.2	−0.06
O/S → C → TI											0.015	0.027	0.55	−0.03	0.06
O/S → R → TI											0.169	0.069	2.467	0.069	0.299
O/S → UWES → TI	−0.4	0.147	2.752	−0.64	−0.16	−0.045	0.107	0.42	−0.22	0.13	−0.2	0.046	4.344	−0.29	−0.13

Note: S/SMT = self-PMIEs compared to SMTs, O/S = other-PMIEs compared to self-PMIEs, O/SMT = other-PMIEs compared to SMTs, A = autonomy satisfaction, C = competence satisfaction, R = relatedness satisfaction, BRN = burnout, UWES = work engagement, DDI = self-disclosure, TI = turnover intentions.

**Table A10:** Moderation Analyses—Direct and Interaction Effects.

Relationship	Path Coefficients	SE	T	95%CI	
				LL	UL
S/SMT → BRN	0.993	0.071	14.029	0.869	1.101
S/SMT → UWES	−1.19	0.098	12.182	−1.349	−1.026
S/SMT → TI	−0.065	0.141	0.461	−0.292	0.172
O/SMT → BRN	0.494	0.054	9.092	0.401	0.579
O/SMT → UWES	−0.487	0.087	5.57	−0.627	−0.339
O/SMT → TI	−0.11	0.107	1.029	−0.283	0.067
O/S → BRN	−0.499	0.059	8.426	−0.592	−0.399
O/S → UWES	0.704	0.074	9.517	0.578	0.823
O/S → TI	−0.045	0.107	0.420	−0.222	0.130
A → BRN	−0.095	0.058	1.622	−0.191	0.002
A → UWES	0.322	0.083	3.863	0.186	0.459
A → TI	−0.235	0.113	2.084	−0.422	−0.049
C → BRN	−0.042	0.021	1.998	−0.076	−0.007
C → UWES	0.056	0.031	1.849	0.007	0.107
C → TI	0.02	0.036	0.556	−0.041	0.078
R → BRN	−0.176	0.058	3.027	−0.27	−0.079
R → UWES	0.339	0.079	4.312	0.211	0.468
R → TI	−0.25	0.091	2.729	−0.401	−0.1
BRN → TI	0.249	0.081	3.066	0.114	0.379
UWES → TI	−0.286	0.059	4.887	−0.383	−0.191
PSS → BRN	−0.053	0.027	1.968	−0.1	−0.01
PSS → UWES	0.021	0.038	0.544	−0.042	0.084
PSS → TI	−0.522	0.054	9.706	−0.609	−0.431
DDI → BRN	−0.347	0.035	9.841	−0.407	−0.291
DDI → UWES	−0.009	0.045	0.199	−0.083	0.066
DDI → TI	0.061	0.056	1.095	−0.028	0.154
PSS × A → BRN	0.012	0.016	0.731	−0.015	0.039
PSS × A → UWES	−0.067	0.023	2.977	−0.105	−0.03
PSS × A → TI	0.03	0.03	0.981	−0.02	0.081
DDI × R → BRN	0.043	0.021	2.039	0.007	0.078
DDI × R → UWES	−0.095	0.029	3.343	−0.142	−0.048
DDI × R → TI	0.07	0.031	2.255	0.019	0.121

Note: S/SMT = self-PMIEs compared to SMTs, O/S = other-PMIEs compared to self-PMIEs, O/SMT = other-PMIEs compared to SMTs, A = autonomy satisfaction, C = competence satisfaction, R = relatedness satisfaction, BRN = burnout, UWES = work engagement, DDI = self-disclosure, TI = turnover intentions.

**Table A11:** Moderation Analyses—Conditional Direct Effects.

Paths	Path Coefficient	SE	T	95%CI	
				LL	UL
A → BRN conditional on PSS at −1 SD	−0.064	0.023	2.797	−0.102	−0.026
A → BRN conditional on PSS at Mean	−0.052	0.018	2.851	−0.083	−0.023
A → BRN conditional on PSS at +1 SD	−0.041	0.026	1.564	−0.084	0.001
A → TI conditional on PSS at −1 SD	−0.16	0.045	3.525	−0.236	−0.086
A → TI conditional on PSS at Mean	−0.13	0.033	3.93	−0.185	−0.076
A → TI conditional on PSS at +1 SD	−0.101	0.044	2.271	−0.172	−0.027
A → UWES conditional on PSS at −1 SD	0.152	0.035	4.339	0.094	0.209
A → UWES conditional on PSS at Mean	0.086	0.028	3.105	0.041	0.131
A → UWES conditional on PSS at +1 SD	0.019	0.036	0.537	−0.04	0.078
R → BRN conditional on DDI at −1 SD	−0.086	0.021	4.12	−0.121	−0.052
R → BRN conditional on DDI at Mean	−0.048	0.019	2.52	−0.08	−0.017
R → BRN conditional on DDI at +1 SD	−0.01	0.032	0.316	−0.063	0.042
R → TI conditional on DDI at −1 SD	−0.104	0.037	2.844	−0.165	−0.046
R → TI conditional on DDI at Mean	−0.042	0.03	1.391	−0.091	0.007
R → TI conditional on DDI at +1 SD	0.02	0.045	0.458	−0.053	0.093
R → UWES conditional on DDI at −1 SD	0.141	0.03	4.772	0.093	0.191
R → UWES conditional on DDI at Mean	0.057	0.027	2.082	0.011	0.101
R → UWES conditional on DDI at +1 SD	−0.027	0.044	0.628	−0.099	0.045

Note: S/SMT = self-PMIEs compared to SMTs, O/S = other-PMIEs compared to self-PMIEs, O/SMT = other-PMIEs compared to SMTs, A = autonomy satisfaction, C = competence satisfaction, R = relatedness satisfaction, BRN = burnout, UWES = work engagement, DDI = self-disclosure, TI = turnover intentions.

**Table A12:** Moderation Analyses—Other Conditional Indirect Effects.

Paths	Path Coefficient	SE	T	95%CI	
				LL	UL
A → BRN → TI conditional on PSS at −1 SD	−0.016	0.008	1.968	−0.033	−0.005
A → BRN → TI conditional on PSS at Mean	−0.013	0.006	2.041	−0.026	−0.005
A → BRN → TI conditional on PSS at +1 SD	−0.01	0.007	1.367	−0.026	−0.001
A → UWES → TI conditional on PSS at −1 SD	−0.044	0.014	3.164	−0.071	−0.025
A → UWES → TI conditional on PSS at Mean	−0.025	0.009	2.7	−0.043	−0.012
A → UWES → TI conditional on PSS at +1 SD	−0.006	0.01	0.535	−0.023	0.011
O/SMT → A → BRN conditional on PSS at −1 SD	0.063	0.024	2.675	0.027	0.105
O/SMT → A → BRN conditional on PSS at Mean	0.052	0.019	2.737	0.024	0.086
O/SMT → A → BRN conditional on PSS at +1 SD	0.04	0.026	1.542	0	0.086
O/SMT → A → TI conditional on PSS at −1 SD	0.158	0.05	3.175	0.083	0.247
O/SMT → A → TI conditional on PSS at Mean	0.128	0.036	3.563	0.074	0.194
O/SMT → A → TI conditional on PSS at +1 SD	0.099	0.045	2.228	0.029	0.176
O/SMT → A → UWES conditional on PSS at −1 SD	−0.15	0.038	3.971	−0.217	−0.093
O/SMT → A → UWES conditional on PSS at Mean	−0.085	0.029	2.94	−0.136	−0.041
O/SMT → A → UWES conditional on PSS at +1 SD	−0.019	0.036	0.534	−0.079	0.038
O/SMT → R → BRN conditional on DDI at −1 SD	0.133	0.034	3.852	0.078	0.191
O/SMT → R → BRN conditional on DDI at Mean	0.074	0.03	2.448	0.026	0.125
O/SMT → R → BRN conditional on DDI at +1 SD	0.015	0.049	0.315	−0.064	0.097
O/SMT → R → TI conditional on DDI at −1 SD	0.16	0.058	2.766	0.071	0.258
O/SMT → R → TI conditional on DDI at Mean	0.064	0.046	1.379	−0.009	0.144
O/SMT → R → TI conditional on DDI at +1 SD	−0.031	0.069	0.456	−0.145	0.082
O/SMT → R → UWES conditional on DDI at −1 SD	−0.217	0.048	4.559	−0.299	−0.142
O/SMT → R → UWES conditional on DDI at Mean	−0.087	0.042	2.063	−0.158	−0.019
O/SMT → R → UWES conditional on DDI at +1 SD	0.042	0.067	0.626	−0.069	0.153
R → BRN → TI conditional on DDI at −1 SD	−0.022	0.009	2.375	−0.04	−0.009
R → BRN → TI conditional on DDI at Mean	−0.012	0.007	1.806	−0.026	−0.004
R → BRN → TI conditional on DDI at +1 SD	−0.002	0.009	0.292	−0.018	0.01
R → UWES → TI conditional on DDI at −1 SD	−0.04	0.013	3.208	−0.065	−0.023
R → UWES → TI conditional on DDI at Mean	−0.016	0.009	1.879	−0.033	−0.004
R → UWES → TI conditional on DDI at +1 SD	0.008	0.013	0.615	−0.011	0.031
S/SMT → A → BRN conditional on PSS at −1 SD	0.117	0.043	2.748	0.047	0.188
S/SMT → A → BRN conditional on PSS at Mean	0.096	0.034	2.799	0.042	0.155
S/SMT → A → BRN conditional on PSS at +1 SD	0.074	0.048	1.555	−0.002	0.155
S/SMT → A → TI conditional on PSS at −1 SD	0.291	0.085	3.412	0.156	0.438
S/SMT → A → TI conditional on PSS at Mean	0.237	0.064	3.736	0.138	0.348
S/SMT → A → TI conditional on PSS at +1 SD	0.183	0.083	2.217	0.051	0.322
S/SMT → A → UWES conditional on PSS at −1 SD	−0.278	0.066	4.239	−0.387	−0.172
S/SMT → A → UWES conditional on PSS at Mean	−0.157	0.052	2.998	−0.244	−0.073
S/SMT → A → UWES conditional on PSS at +1 SD	−0.035	0.066	0.533	−0.146	0.07
S/SMT → R → BRN conditional on DDI at −1 SD	0.074	0.022	3.443	0.042	0.113
S/SMT → R → BRN conditional on DDI at Mean	0.041	0.018	2.346	0.015	0.073
S/SMT → R → BRN conditional on DDI at +1 SD	0.009	0.027	0.314	−0.036	0.055
S/SMT → R → TI conditional on DDI at −1 SD	0.089	0.034	2.617	0.04	0.152
S/SMT → R → TI conditional on DDI at Mean	0.036	0.026	1.351	−0.004	0.083
S/SMT → R → TI conditional on DDI at +1 SD	−0.018	0.039	0.452	−0.083	0.045
S/SMT → R → UWES conditional on DDI at −1 SD	−0.121	0.031	3.934	−0.178	−0.076
S/SMT → R → UWES conditional on DDI at Mean	−0.049	0.024	2.014	−0.091	−0.011
S/SMT → R → UWES conditional on DDI at +1 SD	0.024	0.038	0.619	−0.037	0.088

Note: S/SMT = self-PMIEs compared to SMTs, O/S = other-PMIEs compared to self-PMIEs, O/SMT = other-PMIEs compared to SMTs, A = autonomy satisfaction, C = competence satisfaction, R = relatedness satisfaction, BRN = burnout, UWES = work engagement, DDI = self-disclosure, TI = turnover intentions.
